# Linking epidemiology and genomics of maternal smoking during pregnancy in utero and in ageing: a population-based study using human foetuses and the UK Biobank cohort

**DOI:** 10.1016/j.ebiom.2025.105590

**Published:** 2025-03-12

**Authors:** Mihail Mihov, Hannah Shoctor, Alex Douglas, David C. Hay, Peter J. O'Shaughnessy, John P. Iredale, Sophie Shaw, Paul A. Fowler, Felix Grassmann

**Affiliations:** a Institute of Medical Sciences, School of Medicine, Medical Sciences & Nutrition, University of Aberdeen, Aberdeen, UK; b Institute of Applied Health Sciences, School of Medicine, Medical Sciences and Nutrition, University of Aberdeen, Aberdeen, UK; c Centre for Regenerative Medicine, Institute for Regeneration and Repair, The University of Edinburgh, Edinburgh, UK; d School of Biodiversity, One Health & Veterinary Medicine, University of Glasgow, UK; e Senate House, University of Bristol, Bristol, UK; f All Wales Medical Genomics Service, Institute of Medical Genetics, University Hospital of Wales, Cardiff, UK; g Institute for Clinical Research and Systems Medicine, Health and Medical University, Potsdam, Germany

**Keywords:** Smoking, Epidemiology, Genetics, Transcriptomics, Survival

## Abstract

**Background:**

Maternal smoking and foetal exposure to nicotine and other harmful chemicals in utero remains a serious public health issue with little knowledge about the underlying genetics and consequences of maternal smoking in ageing individuals. Here, we investigated the epidemiology and genomic architecture of maternal smoking in a middle-aged population and compare the results to effects observed in the developing foetus.

**Methods:**

In the current project, we included 351,562 participants from the UK Biobank (UKB) and estimated exposure to maternal smoking status during pregnancy through self-reporting from the UKB participants about the mother's smoking status around their birth. In addition, we analysed 64 foetal liver transcriptomic expression datasets collected from women seeking elective pregnancy terminations. Foetal maternal smoking exposure was confirmed through measurement of foetal plasma cotinine levels.

**Findings:**

Foetal exposure to maternal smoking had a greater impact on males than females, with more differentially expressed genes in liver tissue (3313 vs. 1163) and higher liver pathway activation. In the UKB, maternal smoking exposure was linked to an unhealthy lifestyle, lower education, and liver damage. In a genome-wide analysis in the UKB, we leveraged the shared genetic basis between affected offspring and their mothers and identified five genome-wide significant regions. We found a low heritability of the trait (∼4%) and implicated several disease-related genes in a transcriptome-wide association study. Maternal smoking increased all-cause mortality risk (Hazard ratio and 95% CI: 1.10 [1.04; 1.16], *P* = 4.04 × 10^−4^), which was attenuated in non-smoking males.

**Interpretation:**

Although male foetuses are more affected than females by maternal smoking in pregnancy, this effect was largely reduced in middle-aged individuals. Importantly, our results highlight that the overall 10% increased mortality due to maternal smoking in pregnancy was greatly attenuated in non-smokers. This study demonstrates the importance of campaigns promoting offspring smoking prevention in families where the parent(s) smoke.

**Funding:**

Funding for this project was provided by the 10.13039/501100000882University of Aberdeen, the Science Initiative Panel of the Institute of Medical Science, the UK 10.13039/501100000265Medical Research Council, the Seventh Framework Programme of the 10.13039/501100000780European Union under Grant Agreement 212885 (REEF), 10.13039/501100023968NHS Grampian Endowments grants and the 10.13039/501100000780European Commission Horizon Europe research grant Agreement 101094099 (INITIALISE).

**Research in context**

**Evidence before this study**

Maternal smoking during pregnancy has been linked to many health issues for the offspring. However, we previously had limited knowledge about which in-utero processes are influenced by maternal smoking and how they translate to diminished health later in life.

**Added value of this study**

Here, we linked data from foetal liver tissues to markers of health and disease in middle aged individuals using high-throughput data. We show that males, especially over 16 weeks of gestation, respond most strongly to maternal smoking in-utero. This apparent sex difference in disease risk and mortality persists throughout life, at least into middle age. We are also the first to report genetic variations associated with maternal smoking by leveraging the shared genetics between affected offspring and their mothers.

**Implications of all the available evidence**

Our results indicate that smoking cessation or, ideally, prevention of smoking initiation in men is crucial to counter the increased mortality due to perinatal maternal smoking. In addition, several genes were identified which increase the propensity for maternal smoking, which highlighted relevant and potentially targetable pathways. These findings can therefore offer ways to decrease the societal burden of maternal smoking in our ageing society.

## Introduction

Maternal smoking during pregnancy is estimated to be around 2% worldwide with 8% for Europe and 6% for the Americas.^1^ Consequently, considerable effort has been made to understand the biological effects and health consequences of maternal smoking during pregnancy for the offspring. Tobacco exposure in utero is linked with increased risk of preterm birth (before 37 weeks' gestation),^2^ still-birth, neonatal mortality, miscarriage, foetal growth restriction,^3^ and infant morbidity.^4^^,^^5^ Indeed, third trimester foetuses of mothers smoking cigarettes during pregnancy have significantly smaller body measurements than those of non-smokers.^3^ Additionally, persistent health consequences relating to the offspring of mothers who smoked during pregnancy include higher risk of developing psychological and neurological disorders,^6^, ^7^, ^8^, ^9^ type II diabetes,^10^^,^^11^ obesity and childhood cancer,^11^ cardiovascular disorders,^12^ smoking as an adult^13^ and detrimental changes to pulmonary function in adulthood.^14^ Maternal smoking effects during pregnancy and the consequences for the offspring (including metabolic syndrome) are at the heart of the Developmental Origins of Health and Disease (DOHaD) concept.^15^ We recently showed perfluoroalkyl substances exposure was associated with metabolic changes in foetal livers.^16^ In contrast, using the same population of foetuses in the current study, we found no association of pregnancy maternal smoking with metabolic changes in the foetal livers suggesting an onset of liver metabolic aspects of metabolic syndrome greater than 19 weeks of gestation. Despite the substantial availability of epidemiological data and counselling strategies, smoking during pregnancy is still a persistent issue among women, especially those with lower education attainment and socioeconomic status.^17^^,^^18^

To fully understand the biological consequences of maternal smoking during pregnancy for future generations, several studies have investigated the molecular, genetic, and epigenetic changes in children born to maternal pregnancy smokers. Indeed, lower IGF-1 levels and thus potentially lower β-cell function as a metabolic health risk for the new-borns of smoking mothers have been reported.^10^ Furthermore, pregnancy maternal smoking is associated with transcriptional changes during embryonic and foetal development. Studies on peripheral blood, cord blood, and placenta have reported more than 200 deregulated genes involved in inflammation, immunity, haematopoiesis, vascularisation, signalling, neurogenesis, neuronal function, and addiction^19^^,^^20^ as well as age dependency in the identified associations.^21^ Additionally, genome-wide changes in methylation patterns persistent through life^12^^,^^22^^,^^23^ and have been reported in relation to pregnancy maternal smoking. Such changes are correlated with pathways involved in nervous system and anatomical development, proliferation, apoptosis, differentiation, and oncogenesis,^22^^,^^23^ as well as indicating sex differences in observed associations.^12^

There is strong evidence to suggest that dysregulation of human foetal liver development has consequences for adult health. One link is through impaired foetal growth,^24^ which can lead to foetal programming of metabolic syndrome in adulthood.^25^ A down-stream consequence of increasing metabolic syndrome levels is increased non-alcoholic fatty liver disease, which affects 17–30% of the population in Western countries^26^ and which also has roots in disturbed foetal growth. Programming of metabolic syndrome may also be partially affected by alterations in steroid enzyme activity^27^ and there is strong evidence for greater risk of foetal programming of dysregulated hepatic lipid metabolism in males.^28^ The link between foetal environment (including maternal smoking and nutritional variation), the liver and adult disease remains poorly understood.^29^ For these reasons we selected the human foetal liver for RNAseq analysis in this study.

To date, comprehensive evaluation of the underlying genetic, environmental, and other risk factors in large collections of individuals related to maternal smoking during pregnancy and its consequences for the ageing population is lacking. Whereas substantial research has been conducted investigating the genetic risk contribution of pregnancy maternal smoking in relation to different diseases for the offspring,^30^, ^31^, ^32^, ^33^ most studies use low numbers of individuals and present maternal smoking during pregnancy as a risk contributor to the trait of interest rather than the trait itself. To increase study size and power we have utilised the UK Biobank cohort, which contains data on more than 500,000 individuals. This enables us to uncover non-modifiable, lifestyle and biochemistry factors associated with exposure to pregnancy maternal smoking. These data, together with results from foetal hepatic RNA-seq studies have been used to generate a better understanding of the biological consequences of in utero smoke exposure for future generations.

## Methods

### Study design and setting

The UK Biobank (UKB) is a large cohort of 500,000 UK participants across 22 centres in Scotland, England and Wales aged 40–69 years. The cohort contains genotype data on 488,377 participants with over 805,426 genetic markers available.^34^ In addition, the UKB includes extensive phenotypic, biochemical and medical records of the participants along with information on lifestyle and socio-demographic factors acquired at recruitment through questionnaires, physical measurements and blood, urine, and saliva samples.^34^

At the University of Aberdeen, we collected a cohort of 80 termination embryos in collaboration with the Aberdeen Pregnancy Counselling Service. In this cohort we ascertained maternal data, medications use, and self-reported number of cigarettes smoked per day for each mother. In addition, the cohort contains liver RNA-seq data from all 80 embryos.

### UK Biobank variables considered

Information on maternal smoking status during pregnancy is based on self-reporting from the participant about their own mother's smoking status around birth of the participant (data field 1787). The deprivation index (data field 22189) is a measurement used for socio-economic classification of individuals within the UK. In accordance with the English Indices of Deprivation 2019, the seven main items considered are income, crime, employment, health, education, barriers to housing and living environment. Educational attainment (data field 26414) was coded as having a university degree vs. any other lower level of education. We defined lack of physical activity (data field 22189) as individuals who exercised less than the moderate/vigorous/walking recommendation.^35^ Risky alcohol consumption (data field 1558) was defined as drinking alcoholic beverages more than three times a week.^36^^,^^37^ All-cause survival (data field 22189) and all-cause mortality (data field 22189) over the five-year follow-up period were considered as the outcome in the calculation of survival likelihood ratios of pregnancy maternal smoking cases versus controls. Finally, as an additional quality control (QC) step, individuals of non-European genetic ancestry, those exhibiting sex chromosome abnormalities, individuals which retracted their consent, and missing pregnancy maternal smoking phenotype have been excluded leading to a final sample size of 351,562 individuals. For the phenome-wide (PheWAS) and genome-wide (GWAS) association studies the sample size was further reduced to 344,524 individuals due to samples failing genotyping QC.^38^

### Human foetal collection

Maternal data, medications use, and self-reported number of cigarettes smoked per day were recorded. Only foetuses from normally progressing pregnancies (determined at ultrasound scan prior to termination) from women over 16 years of age, and between 11 and 19 weeks of gestation, were collected following termination by RU-486 (Mifepristone) treatment (200 mg) and prostaglandin induced delivery, as detailed previously.^39^ Foetuses were transported to the laboratory within 30 min of delivery, weighed, sexed and their crown-rump length recorded. Foetal tissues were snap-frozen in liquid-nitrogen, and stored at −80 °C, or fixed in 10% neutral buffered formalin. Pregnancy maternal smoking status was confirmed by measurement of foetal plasma cotinine using a commercially available kit (Cozart Plc, Abingdon, Kent, UK). Cohort data for the 80 foetuses used for RNA-seq are given in Supplementary Table S1. This cohort was balanced for foetal sex and foetal age as far as possible at the time.

### Ethics

Women seeking elective terminations of pregnancy were recruited with full written, informed consent by nurses working independently of the study at Aberdeen Pregnancy Counselling Service. The collection of foetal material involved in the hepatic studies was approved by the National Health Service (NHS) Grampian Research Ethics Committees (REC 04/S0802/21) and the study was conducted according to the guidelines in the Declaration of Helsinki. The current study was conducted under the UK Biobank project 73446.

### Statistics

The association of pregnancy maternal smoking with modifiable and non-modifiable life factors, and immunological markers was explored with logistic (binary markers such as university degree) or linear regression (linear markers such as body mass index) as implemented in core R with the function ‘*glm*’ (R version 4.0.3).^40^ We coded the binary maternal smoking during pregnancy variable (i.e., whether their mother smoked around birth) as the exposure and the respective marker as outcome. Lifestyles factors were investigated in a multivariate linear model including all predictors while blood markers were individually added to the model with all lifestyle factors. Continuous variables in the association analyses were normalised to have a mean of 0 and a standard deviation of 1. Thus, we report the effect sizes per one standard deviation of a marker. Correlation plots were produced using R package ‘*corrplot*’ (version: 0.93).^41^ In order to estimate the effect of pregnancy maternal smoking status on mortality, the cohort was split into eight different groups based on sex and smoking status. A Cox proportional hazards model was applied to each subgroup through R function ‘*coxph*’ available as part of the ‘*survival*’ package (version: 3.3-2).^42^ The Cox proportional hazards model assumptions were investigated using R function ‘*cox.zph*’ available as part of the R ‘*survival*’ package. We observed no significant deviation from those assumptions for maternal smoking after accounting for multiple testing. The results were plotted as a forest plot using R package ‘*metafor*’ (version: 3.0-2).^43^ All survival analyses have been adjusted for sex, BMI, age at baseline (recruitment), individual smoking status (pack years), high alcohol consumption, education, lack of physical activity and deprivation index, red blood cells, lymphocytes, apolipoprotein B (ApoB), cystatin, haemoglobin A1c (HbA1c), insulin like growth factor (IGF), and vitamin D (VitD) concentrations in blood. All adjustments were based on the association results for lifestyle factors and immunological markers as well as their well understood contribution to disease and mortality. Missing data was assumed to be missing at random and thus imputed to the median of all non-missing values.

### Phenome-wide association study of maternal smoking during pregnancy

The UKB cohort was split into two groups (female non-smokers and male non-smokers) aiming to see the effect of the exposure more clearly independent of the smoking status of the participants. Diseases status for the participants has been acquired from the UKB medical records (hospital inpatient data) and was mapped to corresponding phecodes with the ‘*createPhenotypes*’ function and the association analysis was performed using the ‘*phewas*’ function, part of the ‘*PheWAS*’ R package (version: 0.99.5-5).^44^^,^^45^ We coded maternal smoking during pregnancy as the exposure and considered only diseases with an incidence rate of at least 500/500,000 post-recruitment. As threshold of significance, Bonferroni *P* < 5 × 10^−5^ was used (i.e., accounting for 1000 tests). In addition to the previously mentioned adjustments (i.e., the same factors and rational as in the mortality analysis), the PheWAS has also been adjusted for the first three genotype principal components to account for potential residual population structure. The results were plotted using function ‘*ggplot*’ function from the R package ‘*ggplot2*’ (version: 3.3.6).^46^

### Foetal liver differential gene expression and pathway enrichment

RNA from 80 terminations (11–19 weeks of gestation) was extracted from snap frozen liver samples using the Qiagen AllPrep DNA/RNA/Protein Mini kit as per manufacturer's instructions. ERCC spike in controls were added to the RNA, and libraries were prepared for sequencing using the Illumina TruSeq stranded mRNA library prep kit following manufacturer's instructions. The libraries were sequenced using the Illumina NextSeq 500 platform producing 75 bp single end reads at the Centre for Genome Enabled Biology and Medicine, University of Aberdeen. Sequencing was repeated for eight replicate batches and raw reads combined at the end for each sample. Quality metrics were assessed using ‘*FastQC*’ (version: 0.11.3) and read filtering was carried out using ‘*TrimGalore!*’ (version: 0.4.0) with a quality cut off >30. ERCC spike in control reads were removed by alignment to the ERCC reference with ‘*Hisat2*’ (version: 2.0.1) and quality assessed with ‘*erccdashboard*’ (version: 1.6.0). Remaining reads were aligned to the human reference genome GRCh38 using ‘*Hisat2*’ (version: 2.0.1). Alignments were processed using ‘*SAMtools*’ (version: 0.1.19) and reads were quantified at gene regions using ‘*featureCounts*’ (part of the sub read version 5.0.1 package) using parameters to divide counts of multi-mapped reads across their respective genes. Foetal liver RNA-seq expression data was normalised using ‘*vst*’ function from package ‘*DESeq2*’ (version: 1.38.3) and used together with matching phenotypic information about gestation stage (weeks of gestation), foetal sex, maternal BMI, and smoking status. Missing maternal BMI values (*n* = 1) were imputed as the median BMI observed in the remaining samples. BMI and age parameters were scaled to have a mean of 0 and S.D. of 1. Matched datasets within each age group according to sex and smoking status were created using R package ‘*MatchIt*’ (version: 4.3.4)^47^ in order to ensure that there was an equal number of male and female cases in each age group. The samples were exact matched based on in-between sample distances calculated through generalised linear models leading to a final sample size for analysis of 64 foetuses (including the BMI imputed sample; Table 1). Finally, the expression data was restricted to coding genes and genes without reads were excluded. The analysis was performed using R package ‘*DESeq2*’ and ‘normal’ log fold change (LFC) shrinkage method was applied to the results to account for the high variance caused by genes with low read counts.^48^ To estimate the overall impact of maternal smoking during pregnancy on foetal expression, we extracted genes with either unadjusted *P*-value <0.05, |LFC| > 0.5 or both and plotted the data with upset plots using R package ‘*ComplexUpset*’ (version: 1.3.3).^49^ Pathway enrichment analysis was performed on the whole transcriptome with the ‘*WebGestaltR*’ (version: 0.4.4)^50^ package using the Gene Set Enrichment Analysis (GSEA) method.^51^ The analysis was conducted for pathways with gene set size between 50 and 1000 and only the top pathways with *P* < 0.05 and FDR < 0.05 were reported. Based on increasing numbers of pregnancy maternal smoking-affected transcripts with gestational age, the foetuses were divided into three groups according to weeks of gestation: early (12–13 weeks, 20 foetuses), mid (14–16 weeks, 24 foetuses), and late (17–19 weeks, 20 foetuses). Finally, significant differential gene expression (DGE) results (adjusted *P* < 0.05 and |LFC| > 0.5) from the foetal livers were further analysed using QIAGEN IPA (QIAGEN Inc., https://digitalinsights.qiagen.com/IPA) (content version: 94302991, Release Date: 2023-05-27) focussing on affected pathways, upstream regulators, and causal networks.

**Table 1:** Baseline characteristics (mean ± S.D.) of the 64 human foetuses used for liver RNAseq, separated by sex and maternal smoking status.

Variable	Female		Male	
	Non-smoker	Smoker	Non-smoker	Smoker
Number of samples (*n*)	16	16	16	16
Average foetal age (S.D.) (weeks of gestation)	15 ± 2.4	14.75 ± 2.2	14.94 ± 2.4	14.94 ± 2.4
Average foetal body weight (S.D.) (g)	93.4 ± 89.2	72.8 ± 55.6	85.7 ± 74.8	93.9 ± 78.4
Average maternal BMI (S.D.)	24.07 ± 3.2	24.86 ± 6.0	23.71 ± 2.8	24.6 ± 6.0

S.D.—Standard deviation.

### Genome-wide association study of maternal smoking during pregnancy

Since we observed differences in the association signals for males and females, we decided to split the UKB cohort individuals into three groups based on sex (overall cohort, males and females) and perform the GWAS in each group separately. The analysis was carried out on genetic data from the 22 autosomes using ‘*regenie*’ (version: 1.0.6.9), which uses mixed effects models to account for relatedness in the samples thus enabling us to include related individuals in the GWAS with the aim to increase power to detect associations.^52^ Although methods for rare mutation imputation are advancing,^53^ calling of such variants is still time consuming and relatively error prone.^54^ As such, in this study we exclude all variants with minor allele frequency less than 1%, minor allele count less than 1000 (‘*regenie*’ step 1) or less than 100 (‘*regenie*’ step 2) as well as samples and variants with more than 10% genotype missingness and significantly deviating from Hardy–Weinberg equilibrium (*P* > 10^−15^). The filtering of samples and variants was performed using ‘*plink2*’.^55^ The GWAS was adjusted for the first 10 principal components of ancestry, sex, age at baseline, BMI, educational attainment, smoking status and lack of physical activity to account for socio-economic and lifestyle differences which could confound the GWAS. The results were output in the form of summary statistics and as a threshold of significance, the canonical Bonferroni genome-wide significance value was used (*P* < 5 × 10^−8^). The genomic inflation factor was calculated using the formula median (χ^2^)/0.454 (i.e., the median of χ^2^ test statistics taken at one degree of freedom). Finally, functional mapping of statistically significant variants was done using FUMA.^56^ Significant SNPs in each locus were defined at a correlation value R^2^ ≥ 0.6 and we highlighted variants with a CADD score ≥12.37 (i.e., the score represents the deleteriousness of the SNP and thus how likely it is to be functional) in the Manhattan plots.

### Estimation of trait heritability

The proportion of phenotypic variation seen in maternal smoking during pregnancy that can be attributed to genetic variation was computed from the GWAS summary statistics through linkage disequilibrium score regression using statistical software ‘*ldsc*’ (version 1.0.1).^57^^,^^58^ The package provides European LD scores pre-computed from the 1000 Genomes database which were used as regression weights in this analysis.

### Transcriptome-wide association study of maternal smoking during pregnancy

Transcriptome-wide association study (TWAS) was performed on the GWAS summary statistics using ‘*FUSION TWAS*’.^59^ Briefly, the algorithm uses reference expression panels (e.g., GTEx data on 72 tissues, available as part of the ‘*FUSION*’ package used in this study) to learn per gene predictive models of expression which are then applied to the dataset of interest and gene-trait correlations are drawn.^60^ The analysis was performed for all three GWAS subsets mentioned previously using the 1000 Genomes LD database as reference. The results were filtered for genes encoding proteins and statistically significant results were considered at Bonferroni genome-wide significance of *P* < 5 × 10^−8^ (|Z-score| > 5.5) for 1,000,000 tests. Plots were produced using R package ‘*ggplot2*’ (version: 3.3.6).^46^

### Cross-examination between differential gene expression and transcriptome-wide association study

Cross-examination of the differential gene expression and TWAS results was performed by examining the overlap between significantly dysregulated foetal genes and TWAS genes associated with pregnancy maternal smoking genetics. We included transcripts with *P* < 0.05 or |LFC| > 0.5 from the differential gene expression analysis and with *P* < 0.01 (|Z-score| > 2.5) from the TWAS. Data was visualised using R package ‘*ComplexUpset*’.

### Role of funders

The funders were not involved in the study design, model evaluation, analysis and interpretation of results or writing of this manuscript.

## Results

### Identification of differentially expressed genes in the foetal human liver

**Fig. 1:**
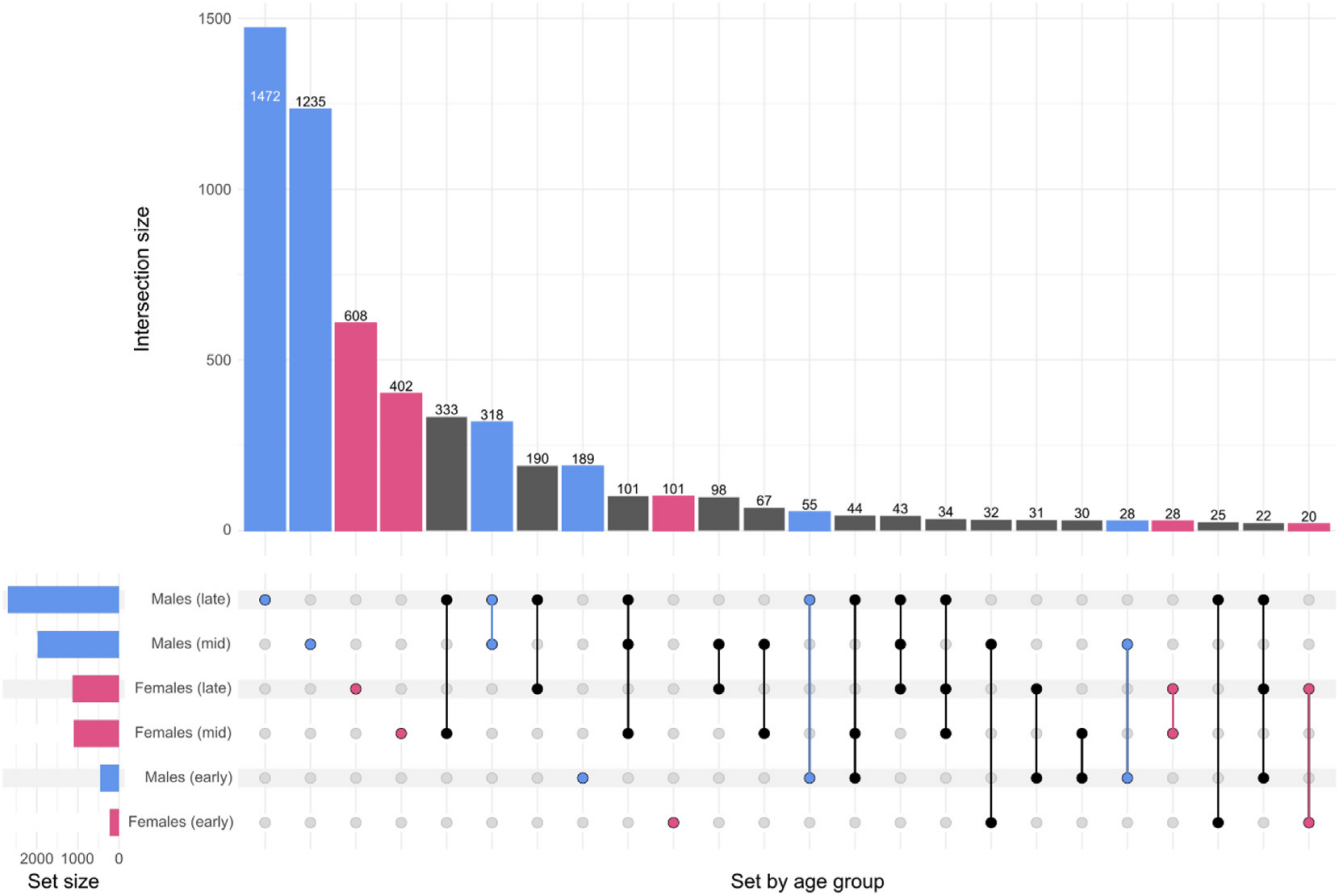
**Upset plot representing the number of unique and overlapping genes in foetal livers from males and females at early (11**–**13), mid (14**–**16), and late (17**–**19) weeks of gestation.** Set size represents the total number of differentially expressed (DE) genes for the subset defined by the gestational week within the second trimester and sex of the foetus. Intersection size represents the number of genes in the corresponding intersection. Connected dots in the intersection indicate overlap in DE genes whereas solo dots indicate intersections with unique DE genes for the corresponding subset. DE genes were considered at *P* < 0.05 or |Log Fold Change| > 0.5. Only intersections with minimum size of 14 are shown.

Differential gene expression analysis was performed on 64 matched foetal livers (Table 1). The number of genes between pregnancy maternal smoking cases and controls with either *P* < 0.05, |LFC| > 0.5 or both at each stage of the analysis are shown in Fig. 1.

**Fig. 2:**
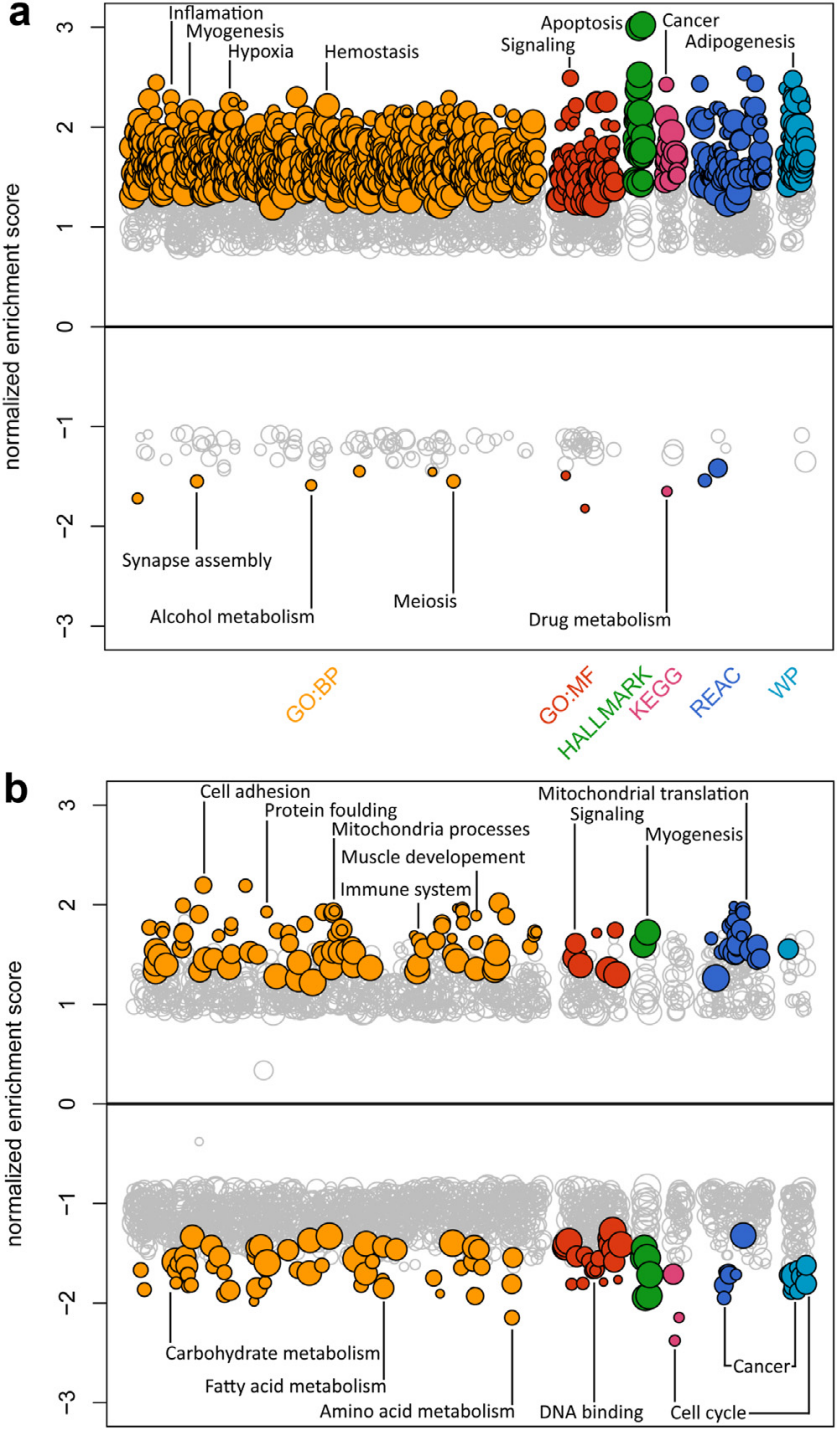
**Mirror plots representing significantly over- and under-represented pathways (indicated by coloured dots) in the DGE in the 17**–**19 weeks of gestation foetal liver for (a) males and (b) females.** The pathways have been analysed in six different databases (GO:BP, GO:MF, HALLMARK, KEGG, REAC, and WP) using the gene set enrichment method. The strength of the association is indicated by the normalised enrichment scores and only the top hits from each panel have been labelled.

Strikingly, we observed that liver gene expression in male foetuses were considerably more affected by maternal cigarette smoking as they had substantially higher numbers of differentially expressed (DEG) genes (3313 vs. 1163) in comparison to the female foetuses, at every stage of the pregnancy studied. Additionally, 1135 genes overlapped between males and females across all stages analysed (Fig. 1). This indicates a potential for common pathways being affected by pregnancy maternal smoking for both sexes. To further dissect the biology at 11–19 weeks of gestation, and to identify any additional pathways that are different for both sexes, we performed a pathway enrichment analysis (Fig. 2). Foetuses demonstrated a substantial sex difference in their respective over- and under-represented pathways with the only similarities being observed for muscle development, cell cycle and signalling. Interestingly, males show an over-representation in pathways involving cancer development whereas those pathways are under-represented in females. We also observed an increase in DEG associated with inflammation, decreased synapse assembly, and alcohol and drug metabolism in males (Fig. 2a), but not in females. In contrast, the number of DEGs associated with mitochondrial processes increased while carbohydrate, fatty and amino acid metabolism decreased in females only.

**Fig. 3:**
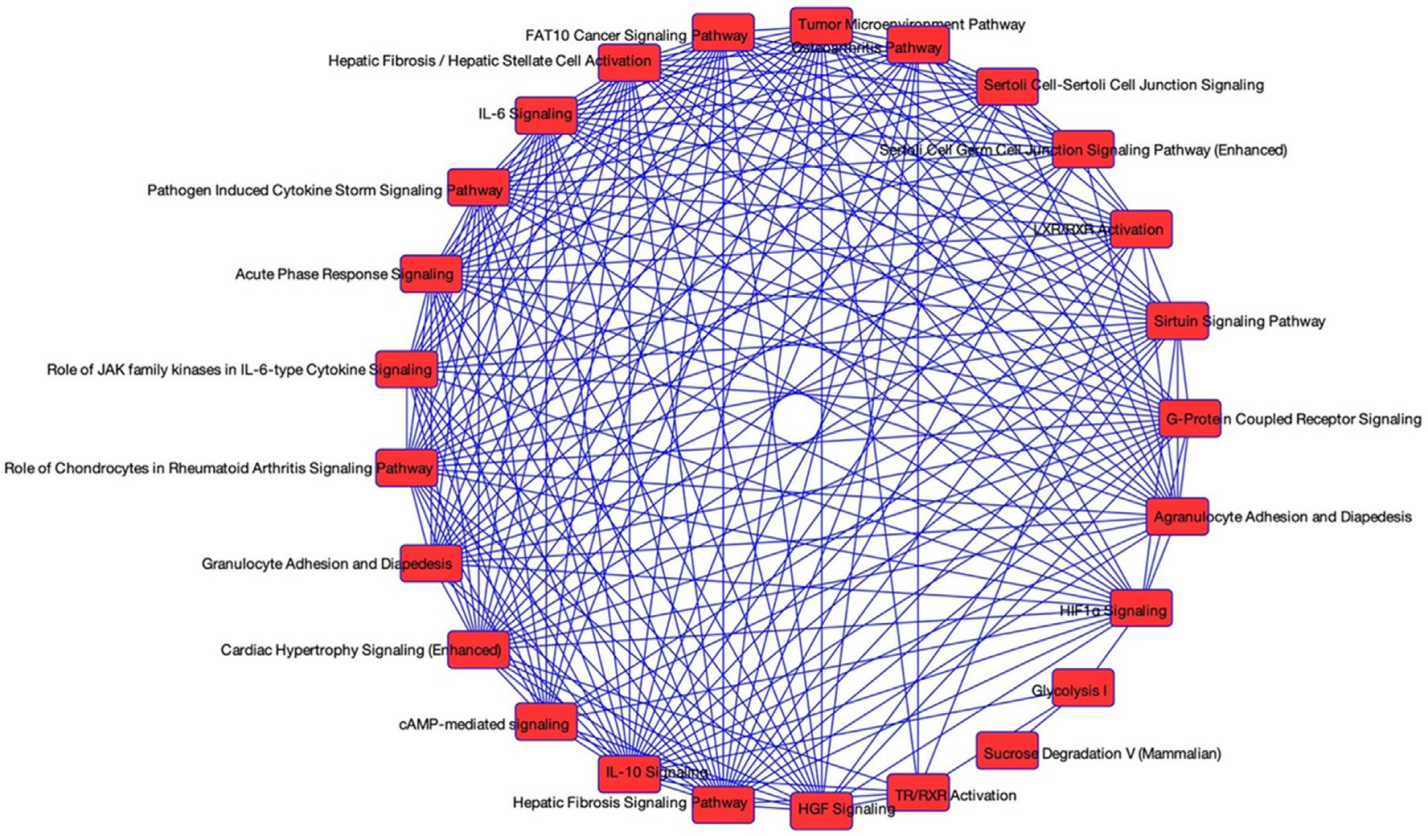
**Overlapping, significant canonical pathways in smoke-exposed 17**–**19 weeks of gestation male foetal livers.** Each pathway is represented as a single node coloured proportionally to the right-tailed Fisher's Exact Test *P*-value, where significance is indicated by the intensity of the red colour. In this analysis all results have been found to be highly significant.

Finally, significant DEG from the foetal livers were further analysed using IPA. Consistent with our previous analyses, the strongest effects were observed in the late second trimester group of males (over 16 weeks of gestation). Strikingly, results were consistent only in the male late study window, with significant z-score and 2-fold change activation/inhibition effects seen, further reflected in significant activation across overlapping canonical pathways (Fig. 3). All further reporting in this section is limited to the male late study window of 17–19 weeks of gestation. Of the top 20 canonical pathways by *P*-value, 12 had positive z-scores, indicative of activation, while only 2 exhibit negative (inhibition) z-scores (Supplementary Figure S1). Of the former, TR/RXR Activation, Tumour Microenvironment Pathway, Acute Phase Response Signalling, HIF1α Signalling, and Hepatic Fibrosis Signalling, amongst others showed activation of most members/processes. For the latter, both Sirtuin Signalling Pathway and LXR/RXR activation pathway did not show a clear majority of pathway members/processes inhibited. Overall, the DEGs in cigarette smoke exposed and non-exposed male foetuses in the 17–19 weeks of gestation group mapped onto 10 main networks (Supplementary Table S2), of which none were directly related to metabolism. Instead, they were associated with development and abnormal development processes, cell death, survival, morphology, and signalling as well as immune response and disease. This range of activities was largely reflected in the more than 2-fold activated/inhibited disease and biological functions (Supplementary Table S3). Next, these data were further explored using IPA's regulator effects (Supplementary Table S4). We found elements of cigarette smoke, such as polycyclic aromatic hydrocarbons (products of combustion) which target the aryl hydrocarbon receptor (AHR) system and of hypoxia which HIF1 response to, amongst others, to frequently appear in the list of regulators. Finally, regarding diseases and functions listed in this table, overall, a wide range of non-metabolic processes were affected although some metabolic processes emerged such as fatty acid metabolism.

### Maternal smoking during pregnancy association with modifiable and non-modifiable lifestyle factors, blood and biochemical markers

**Fig. 4:**
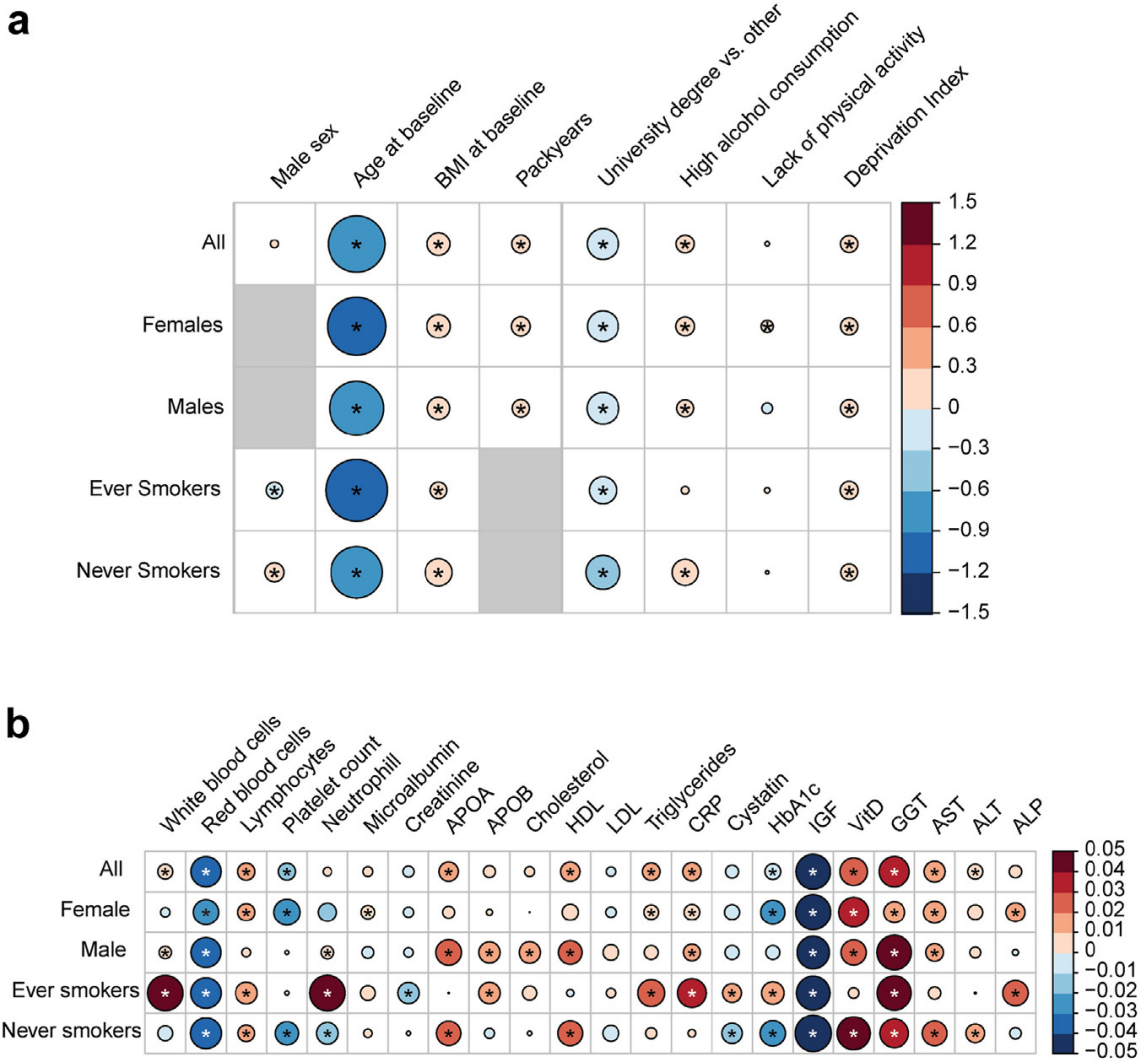
**Association of maternal smoking during pregnancy modifiable and non-modifiable lifestyle factors in adult offspring (a) and blood and biochemistry markers (b).** Briefly, the binary maternal smoking during pregnancy variable was coded as the exposure and the respective marker as outcome. In (a), the results from multivariate linear regression models including all markers are shown for each subgroup. In (b), each marker was added individually to the model from the respective lifestyle analysis to generate the observed associations. The size and colour of the circle indicates the magnitude and direction of the association, respectively. Statistical significance is indicated by an asterisk. Blood and biochemistry markers have been normalised to have a mean of zero and standard deviation of one. BMI—body-mass index; APOA/B—apolipoprotein A/B; H/LDL—high/low density lipoprotein; CRP—C-reactive protein; HbA1c—haemoglobin A1c; IGF—insulin-like growth factor; VitD—vitamin D; GGT—gamma-glutamyl transferase; AST—aspartate transferase; ALT—alanine transaminase; ALP—alkaline phosphatase.

To investigate the potential long-term epidemiological consequences of gestational maternal smoking on the health of individuals aged 40–69 years, we utilised phenotypic data on 351,562 individuals passing quality control from the UKB cohort (Table 2). First, we tested the pregnancy maternal smoking phenotype for association with well-established risk factors for many major age-related diseases such as age, sex, BMI, pack years, high alcohol consumption, education, lack of physical activity, and deprivation index (Fig. 4a). We observed a weak positive correlation between male sex and maternal smoking during pregnancy as well as a negative correlation with age at baseline suggesting that males and younger individuals in general are more likely to report that their mothers smoked while pregnant. Additionally, the positive correlation between pregnancy maternal smoking and offspring pack years agrees with previous research suggesting that the children of maternal smokers are more likely to smoke themselves. Similarly, positive correlations were also observed for higher BMI, excessive alcohol consumption and deprivation index, a unit measurement of socio-economic status, which is supported by the observed strong negative correlation with education status. Interestingly, although lack of physical activity showed no significant association with maternal smoking during pregnancy, when restricted to females we found that exposure to maternal smoking during pregnancy was associated with reduced physical activity.

**Table 2:** Baseline characteristics of UK Biobank participants by sex and smoking status.

Variable	UK Biobank participants				
	Overall	Males	Females	Smokers	Non-smokers
Number of individuals [cases/controls]	108,166/243,396	50,792/109,343	57,374/134,053	49,275/106,110	58,569/136,485
Average age (S.D.) [years]	56.8 (8.0)	57.0 (8.1)	56.5 (8.0)	57.6 (7.9)	56.1 (8.1)
Average body mass index (S.D.) [kg/m^2^]	27.4 (4.7)	27.8 (4.2)	27.0 (5.1)	27.7 (4.7)	27.1 (4.7)
Average pack years smoked (S.D.)	8.2 (15.7)	10.7 (18.5)	6.1 (12.6)	23.4 (18.7)	0.0 (0.0)
Education level [*n* (%)]	111,257/38.5%	53,403/40.5%	57,854/36.8%	41,994/34.5%	69,093/41.4%
High alcohol consumption [*n* (%)]	159,585/47.0%	86,173/55.6%	73,412/39.8%	81,046/54.4%	78,073/41.2%
Physical activity levels [*n* (%)]	51,931/18.1%	25,128/18.3%	26,803/17.8%	23,463/18.5%	28,330/17.7%
Average deprivation index (S.D.)	−1.6 (2.9)	−1.6 (3.0)	−1.6 (2.9)	−1.2 (3.1)	−1.9 (2.7)

S.D.—Standard deviation.

Maternal smoking during pregnancy could also influence offspring organ function on a systemic level. Therefore, we also tested the exposure for associations with blood cell counts as well as 16 biochemistry markers involved in liver, kidney and cardiovascular function, inflammation and cholesterol, calcium and phosphate metabolism (Fig. 4b). We observed a mild positive correlation between maternal smoking during pregnancy and both white blood cell and neutrophil levels. Conversely, we observed a strong negative correlation with red blood cell counts across all subsets and mild negative correlation with platelet counts when stratified by sex. Finally, markers of lipid metabolism (ApoB and HbA1c), liver function (cystatin), calcium and phosphate (VitD) metabolism, growth hormone signalling (IGF-1) as well as liver function (Gamma-glutamyl Transferase (GGT) and aspartate aminotransferase (AST)) were significantly associated with pregnancy maternal smoking. Since the biochemical markers and the immune system are well understood for their role in health and disease, they could impose potential confounding effects on subsequent epidemiological analyses and have been adjusted for.

### Phenome-wide association study of maternal smoking during pregnancy in non-smoker males and females

**Fig. 5:**
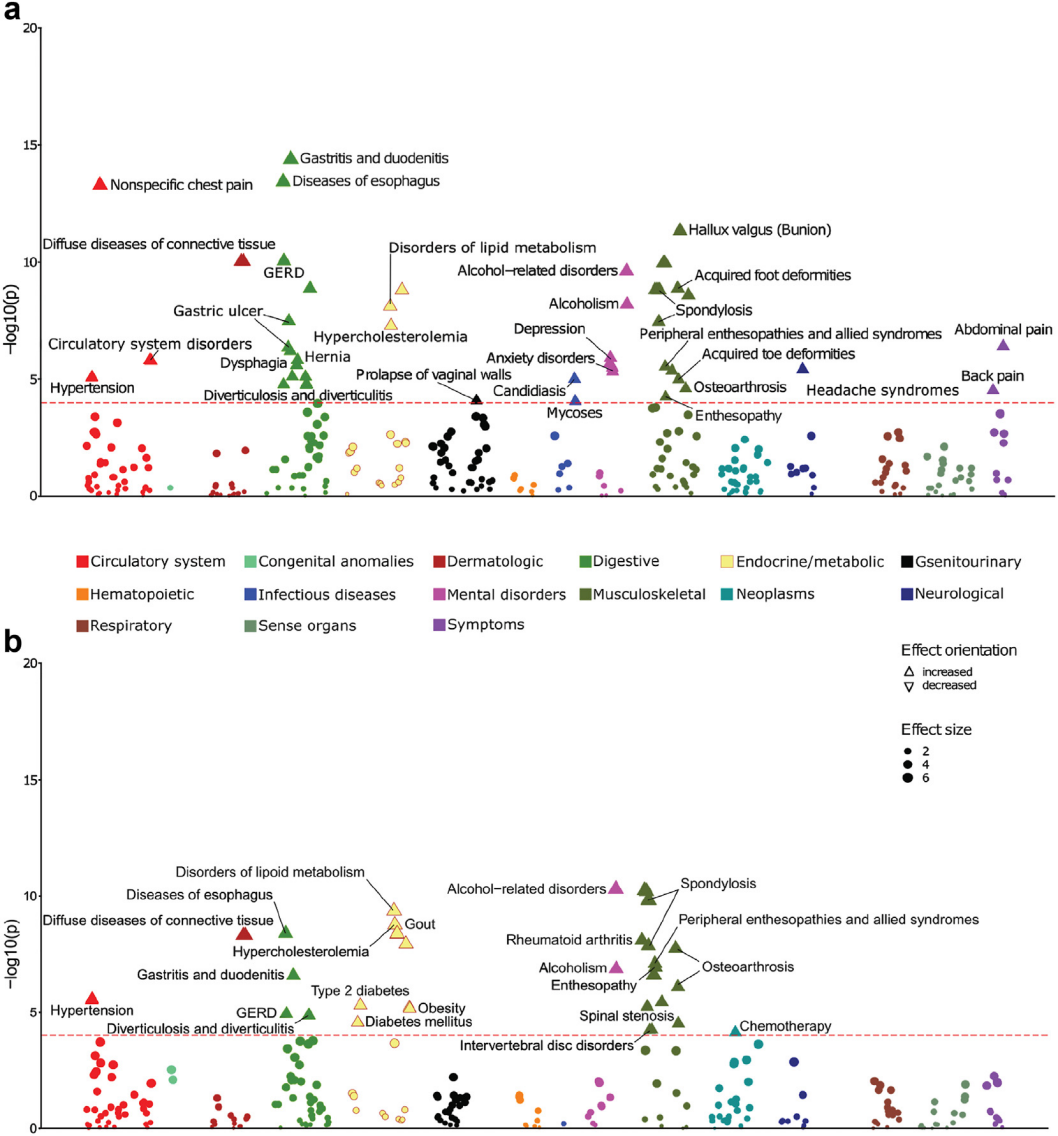
**Phenome-wide association study of maternal smoking during pregnancy in adult offspring non-smoking (a) females and (b) males.** The health consequences of cigarette smoke exposure in the womb were evaluated over 1850 disease phenotypes from the UK Biobank medical records for all non-smoker UK Biobank participants. Associations were drawn using logistic regression models adjusted for age at baseline, BMI, alcohol consumption, education, lack of physical activity, deprivation index, the first three genotype principal components and levels of red blood cells, lymphocytes, apolipoprotein B, cystatin, haemoglobin A1c, insulin like growth factor and vitamin D concentrations in blood. Only diseases occurring in at least 500 individuals post recruitment were considered. Significant associations are depicted above the red line, which represents negative log10 scale of the Bonferroni adjusted *P*-value cut-off for 1000 tests. Direction of the triangles indicates increased (up) or decreased (down) risk for acquiring the disease.

To evaluate the long-term health consequences of maternal smoking during pregnancy for the adult offspring, we performed a Phenome-wise association study (PheWAS) between the exposure and the UKB medical records over a total of 1850 disease phenotypes. Since smoking status has a strong effect on disease incidence even after being adjusted for, we chose to consider only self-reported non-smokers aiming for clarity of the disease-exposure associations. Additionally, building on our previous observations of sex differences during pregnancy, we considered males and females separately in this analysis (Fig. 5). We found exposure to cigarette smoke in-utero for women to be significantly associated with an increased risk of heart and digestive diseases, vaginal wall prolapse, hernia, depression and anxiety, mycoses, headache syndromes, back and abdominal pain. In contrast, men had an increased risk of gout, type II diabetes, and cancer (i.e., measured by prescription of chemotherapy), which were not observed in women (Fig. 5).

### Dissecting long-term heritable consequences for the offspring of pregnancy maternal smokers

**Fig. 6:**
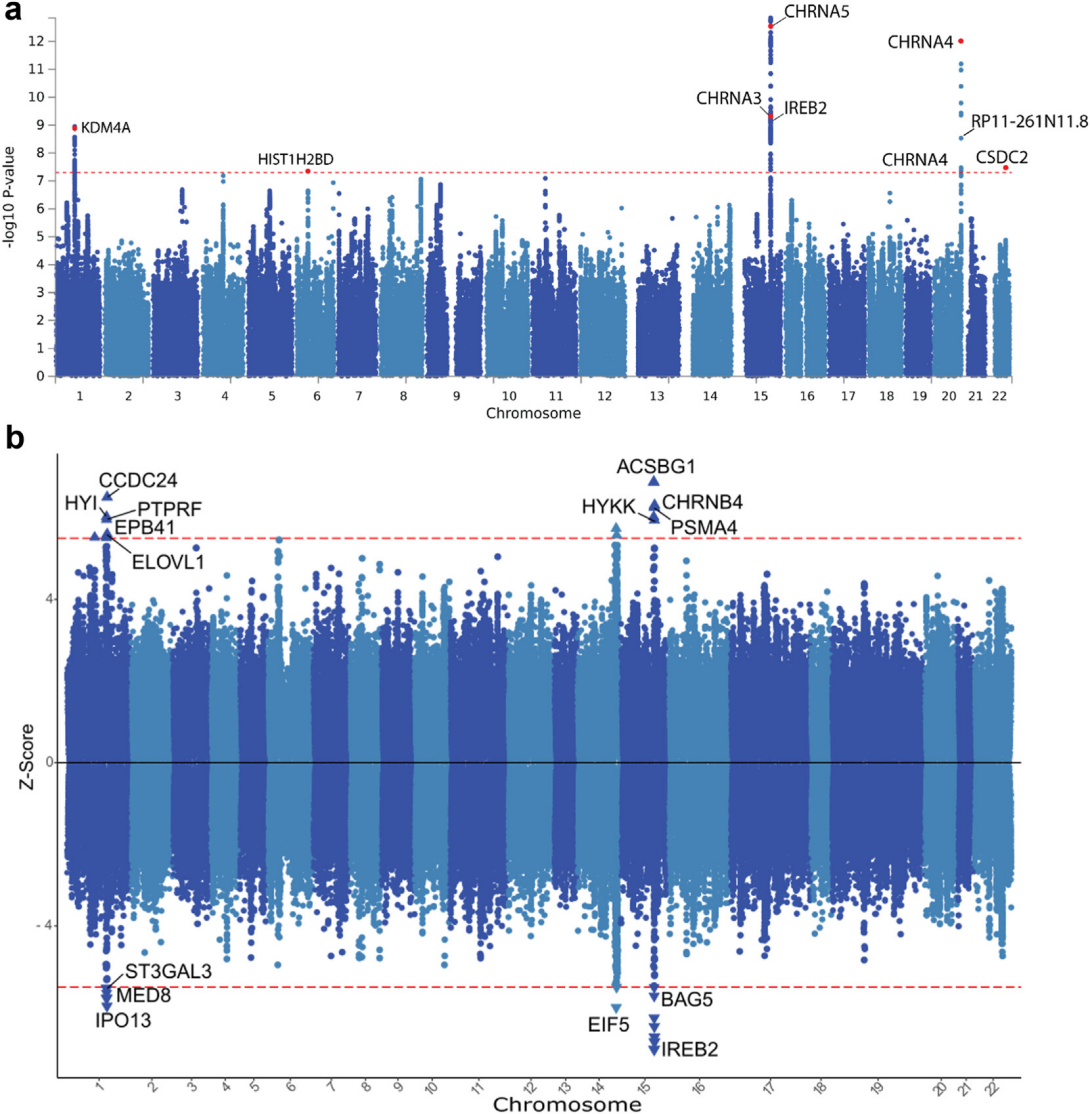
**Summary of the genome-wide (a) and transcriptome-wide (b) association studies of maternal smoking during pregnancy for the whole adult offspring cohort.** (a) Manhattan plot of 4,632,342 short nucleotide polymorphisms with minor allele frequency of at least 1%. Only genes harbouring exonic variants have been labelled. Red dots indicate genes in each region harbouring a statistically significant variant which has a CADD score greater than 12.37, indicating it is likely influencing that gene's function. The red line denotes genome-wide significance (*P* < 5 × 10^−8^, logistic regression) for 1,000,000 tests. The estimated genomic inflation factor λ was 1.256. (b) Manhattan plot representing the association of GWAS variants with GTEx expression panels from 72 tissues. Only known coding genes reaching statistical significance are labelled. The red line denotes Bonferroni genome-wide significance (*P* < 5 × 10^−8^; |Z-score| > 5.5, logistic regression) for 1,000,000 tests. Direction of the triangles indicates genes predicted to be overexpressed (facing up) or underexpressed (facing down).

Ideally, to understand the genetic basis of maternal smoking during pregnancy, a genome-wide association study should be conducted in mothers who reported that they smoked during pregnancy or where pregnancy maternal smoking was established by a blood test in the offspring. However, here we utilise the offspring of mothers who smoked during pregnancy as they share 50% of the genetic makeup of their mother. Thus, this allows us to investigate the genetics of maternal smoking during pregnancy in all UKB individuals, which passed quality control (Fig. 6, Supplementary Figure S2a).

To this end, a total of 4,632,342 variants were tested for association with the trait. We uncovered five genome-wide regions harbouring nine exonic variants reaching genome-wide significance (*P* < 5 × 10^−8^, logistic regression, Fig. 6a). We found genes related to nicotine addiction (*CHRNA3, CHRNA4, CHRNA5*), neurodegeneration (*IREB2*), chromosome maintenance (*HIST1H2BD*), mRNA stability (*CSDC2*), and transcriptional repression to be potentially affected by the pregnancy maternal smoking phenotype. To identify any additional signals that could have been potentially confounded by sex or smoking status, we analysed females, males, smokers, and non-smokers separately (Supplementary Figures S2b–e and S3). While females retained two of the signals in the main analysis (*CHRNA5* and *IREB2*, Supplementary Figure S3a), males had a single new signal related to a gene involved in spinal muscular atrophy (*FGFBP3*, Supplementary Figure S3b). Additionally, two new signals in genes related to fatty acid metabolism (*FADS2*) and signalling (*PTPRF*) were identified in the never-smoking cohort (Supplementary Figure S3d). To estimate the heritability of the pregnancy maternal smoking phenotype attributed to genomic variation, we used a linkage disequilibrium score regression (LDSC) approach over all GWAS subsets. We estimated a moderate SNP-heritable component of 3–4% for pregnancy maternal smoking which indicates the observed effects of maternal smoking during pregnancy in the UK Biobank are mostly epigenetic.

To gain further insight into the potential biological mechanisms underlying SNPs associated with maternal smoking during pregnancy, we investigated the GWAS summary statistics data for variants involved in modulating gene expression. We performed a transcriptome-wide association study (TWAS) using GTEx data on 72 tissues (Fig. 6b). Subsequently, we identified 15 differentially expressed protein coding genes (significance threshold *P* < 10^−8^, |Z-score| > 5.5, logistic regression) associated with the pregnancy maternal smoking phenotype. These include genes involved in neurodegeneration (*EIF5*), diseases of the central nervous system (*IPO13*, *ST3GAL3*), tobacco addiction (*CHRNB4*, *PSMA4*), fatty acid metabolism (*ACSBG1*), and skin conditions (*HYI*, *ELOVL1*). We further investigated the different cohort groups separately to identify any between-group differences (Supplementary Figure S4). We observed sex and smoker status differences in the number of identified genes. Interestingly, three of the genes identified in the GWAS were also identified in the TWAS (*IREB2*, *PTPRF,* and *FADS2*; Fig. 6b, Supplementary Figure S4a and d). Finally, to identify any genetic changes persistent through life we performed cross-examination of the results generated by the DGE and overall TWAS (Supplementary Figure S5a). We note that male foetuses had higher degree of overlap with the TWAS at every stage of development studied than female foetuses. This observation held largely true when only liver specific TWAS signals were considered (Supplementary Figure S5b).

### Maternal smoking during pregnancy effects on individuals' survival likelihood over five years of follow-up

**Fig. 7:**
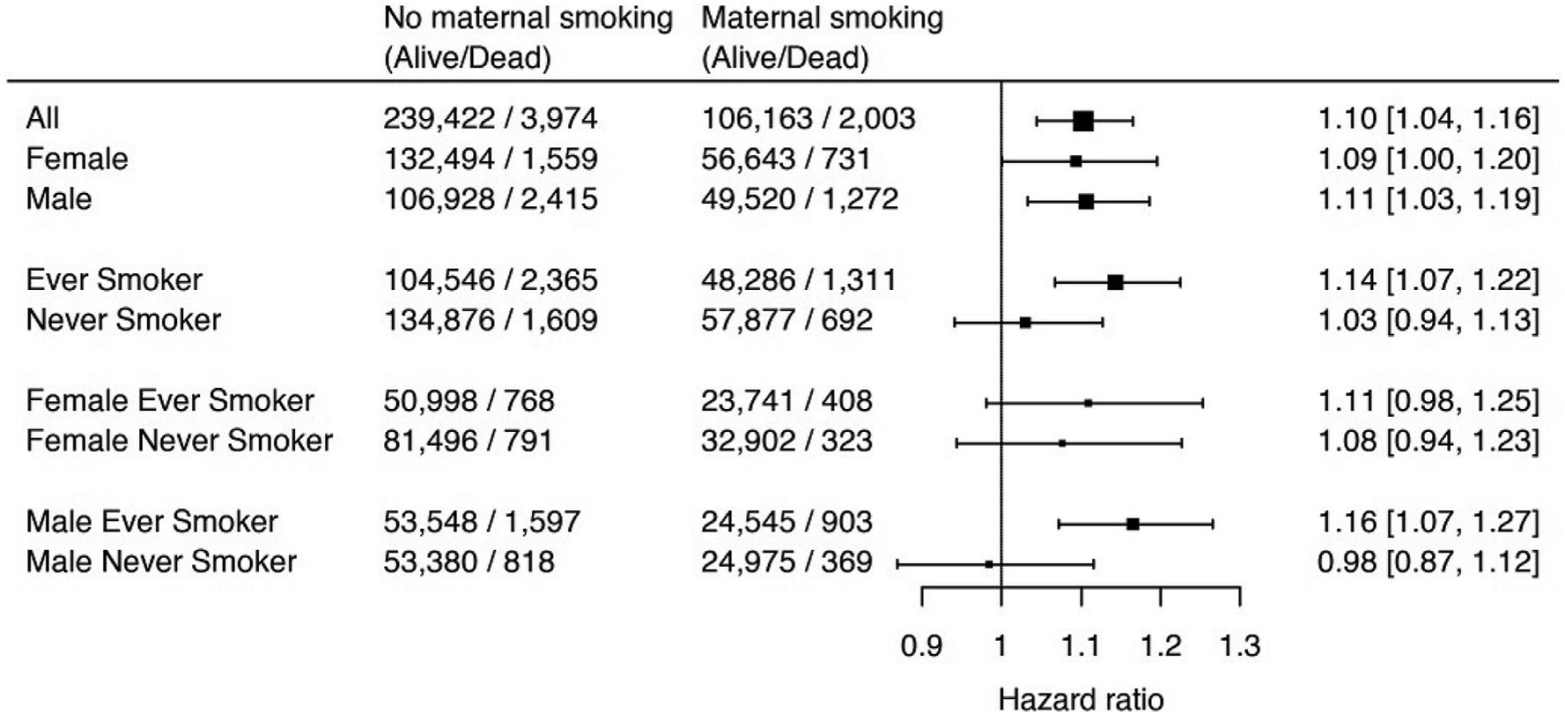
**Forest plot representing the all-cause hazard ratio of maternal smoking during pregnancy status over 5 years of follow-up.** Cases represent individuals who reported that their mothers smoked during pregnancy as opposed to controls. The analysis has been adjusted for sex, BMI, age at baseline, individual smoking status (pack years), high alcohol consumption, education, lack of physical activity and deprivation index and levels of red blood cells, lymphocytes, apolipoprotein B, cystatin, haemoglobin A1c, insulin like growth factor and vitamin D concentrations in blood. Baseline hazard ratio threshold is indicated by a dotted line. Estimate indicates the hazard ratio for the subgroup. CI indicates confidence interval.

To evaluate the effects of maternal smoking during pregnancy on an individual's survival likelihood, we fitted a Cox proportional hazards model to estimate the odds ratio of premature death as a consequence of maternal smoking during pregnancy. The analysis was stratified for sex, smoking status, and a combination of both at five years follow-up (Fig. 7). Our observations indicate a 10% higher risk of dying from all causes due to pregnancy maternal smoking in all individuals (Hazard ratio (HR) and 95% confidence intervals (CI): 1.10 [1.04; 1.16], *P* = 4.4 × 10^−4^, Cox regression). Within individuals that reported ever smoking, mortality was increased due to maternal smoking during pregnancy (Hazard ratio and 95% CI: 1.14 [1.03; 1.19], *P* = 1.3 × 10^−5^, Cox regression) as opposed to much lower risk for never-smokers (Hazard ratio and 95% CI: 1.03 [0.94; 1.13], *P* = 0.52, Cox regression). To evaluate any sex specific differences, we considered males and females separately. On average, maternal smoking during pregnancy has similar effect on the mortality of women (Hazard ratio and 95% CI: 1.09 [1.00; 1.20], *P* = 0.049, Cox regression) compared to men (Hazard ratio and 95% CI: 1.11 [1.03; 1.19], *P* = 4.0 × 10^−3^, Cox regression). Interestingly, when further stratifying sex specific analyses by smoking status, we observed a similar risk in female smokers and non-smokers (HR 1.11 and 1.08, respectively). In contrast, the risk of dying due to maternal smoking in pregnancy was 16% higher in male smokers (Hazard ratio and 95% CI: 1.16 [1.07; 1.27], *P* = 3.1 × 10^−4^, Cox regression), but was almost completely attenuated in men who never smoked (Hazard ratio and 95% CI: 0.98 [0.87; 1.12], *P* = 0.81, Cox regression).

## Discussion

In this study, we investigated the epidemiological and molecular basis of maternal smoking during pregnancy. The effects on offspring were then studied, utilising data from foetal human livers and middle-aged adults from the UK Biobank. We observed a strong sex bias in dysregulated gene expression in foetuses by just under five months of pregnancy (19 weeks of gestation) with males more affected than females. Additionally, in adult offspring, we noted a strong association of maternal smoking during pregnancy with biochemistry markers linked to liver and kidney function. Furthermore, we demonstrated sex differences in disease associations in relation to maternal smoking during pregnancy, with men more likely to develop gout, type II diabetes, become obese or suffer cancer and undergo chemotherapy. Whereas women had increased tendency for mycoses, back and abdominal pain, gastroesophageal reflux disease (GERD), anxiety, depression, and headache syndromes. Our genetic dissection of maternal smoking during pregnancy estimated a low heritability of 3–4% and further functional analysis of the associated SNPs identified genes involved in nicotine addiction, fatty acid metabolism, and neurodegeneration. Finally, we observed a difference in mortality due to sex and smoking status in adult offspring that associated with maternal smoking during pregnancy. Importantly, the excess risk due to pregnancy maternal smoking was reduced in males that had never smoked, but not in females.

To uncover the social and personal impact of maternal smoking during pregnancy, we investigated its association with modifiable and non-modifiable lifestyle factors. We observed a negative correlation of maternal smoking during pregnancy with higher education attainment and age of participants at recruitment and a positive correlation with poor socioeconomic status (indicated in this study as deprivation index), which is in agreement with previous studies.^1^^,^^4^^,^^17^ Indeed, previous reports indicated a decline in maternal smoking during pregnancy in recent years in high income countries as well as personal socioeconomic status and education^1^^,^^4^^,^^17^ which agrees with our observed associations. Additionally, persistent exposure to tobacco smoke in-utero correlates with early onset (age 15) of smoking^13^ in children and increased tendency for psychiatric disorders and substance abuse (e.g., alcohol),^6^ which further supports our observations of positive correlations with smoking status and high alcohol intake in middle-aged adults. Maternal smoking during pregnancy is associated with low birthweight,^61^ indicative of malnutrition during development, with a rapid growth compensation phase during infancy, predominantly seen in males, leading to obesity and overweight.^62^ A positive association between high BMI and pregnancy maternal smoking has been demonstrated previously for the period between early childhood and early adulthood (aged 3–33).^63^ In our study, we observed a similar association in individuals aged 40–69 years as well as potential perturbation to the lipid metabolism for both sexes as indicated by the DGE pathway enrichment analysis. Interestingly, our results indicated a positive association between maternal smoking during pregnancy and male offspring sex. It is, however, not clear why male UK Biobank participants are more likely than female participants to indicate that their mothers smoked during pregnancy. Since there were no further questions regarding pregnancy maternal smoking status in the UK Biobank questionnaire, we cannot speculate on the precise mechanism behind this observation at present but in our foetal studies we do not observe a sex difference associated with validated pregnancy maternal smoking with equal numbers of male and female foetuses in smoking pregnancies across 20 years of observation. The negative association of age at recruitment and pregnancy maternal smoking is, however, quite striking. The association would indicate that younger UK Biobank participants (independent of sex, smoking status or education level) are more likely to report pregnancy maternal smoking than older participants. This could be due to a heightened awareness of younger participants about the adverse effects of maternal smoking during pregnancy as a consequence of smoking prevention strategies.

To gain further insight into the biological basis of maternal smoking during pregnancy associated effects in adulthood, we investigated normalised counts of blood cells and biochemistry markers from whole blood. We observed a strong positive correlation to white blood cells and a strong negative correlation to red blood cells. Higher levels of white blood cells are a hallmark for inflammation,^64^ which is supported by the observed increased levels of C-reactive proteins in individuals exposed to maternal smoking during pregnancy. Pertinently, inflammation is among the leading consequences of obesity which in turn leads to a variety of metabolic diseases such as type II diabetes, hypertension, cardiovascular disease, cancer, neurodegeneration, osteoarthrosis, liver damage, and others.^65^ Furthermore, inflammation pathways were also identified in our DGE pathway enrichment analysis. Interestingly, we found lower IGF levels in middle-aged individuals exposed to maternal smoking during pregnancy. Generally, babies born to women who smoked during pregnancy have a lower birth weight and reduced foetal and adolescent growth.^66^ Indeed, lower levels of IGF-1 were observed in umbilical cord blood if the mothers smoked during pregnancy^67^ and this effect seems to be persistent even four decades later in life. Remarkably, we found a strong positive correlation between pregnancy maternal smoking status and GGT levels, across all considered groups. High concentrations of GGT correlate with an increased risk for liver cirrhosis, cardiovascular disease, diabetes, and cancer.^68^ Considering our observations and since there is a strong interplay between these variables in modulating disease prevalence, they were used as adjustment in our PheWAS analysis.

Smoking has a strong association with inflammation and thus a range of different diseases.^69^ As such, combined with our observations of a strong association between pregnancy maternal smoking status and sex bias in relation to reported pregnancy maternal smoking, we decided to perform our PheWAS on non-smokers only for both sexes separately. The aim was to eliminate potential confounding on the observed disease associations, allowing us to examine the burden of maternal smoking during pregnancy more clearly on the health of future generations. We observed the risk for hypertension and alcoholism to be increased similarly for both sexes which is in line with our previous observations on lifestyle and blood marker associations and with prior research.^6^^,^^65^ Additionally, women were found to be at higher risk of depression and anxiety as well as GERD which was previously reported to be marginally higher in women compared to men.^70^ Conversely, we observed a higher risk for type 2 diabetes, obesity, and cancer (chemotherapy) for men only, which together with our results from the DGE pathway enrichment analysis, is potentially indicative of persistent lifelong changes and a role of maternal smoking during pregnancy in modulating risk through previously unexplored mechanisms.

In order to untangle any potential persistent pathway perturbations acquired during development we utilised IPA to probe our foetal data, which resulted in reinforced marked sex and developmental stage differences. These findings are consistent with the findings in older men who had smoked themselves, indicating dysregulation in immune, developmental and metabolic function in association with maternal smoking during pregnancy. Although metabolic changes associated with maternal smoking in the foetuses were limited, as recently confirmed by us,^16^ we have previously reported sex and pregnancy maternal smoking associated differences in the foetal liver and the thyroid, however, the mechanisms have not been elucidated.^71^^,^^72^ Since it was only the 17–19 weeks of gestation males in the present study who displayed marked hepatic gene pathway consequences associated with maternal smoking during pregnancy, this suggests the male late study window foetuses were experiencing adverse effects on liver development and possibly maladaptive hepatic responses to the toxic chemicals in cigarette smoke. The activation of a range of pathways, such as Acute Phase Response, in association to maternal smoking during pregnancy demonstrated a potential defencive response to inflammation, as is seen in second hand smokers.^73^ Overall, when exploring disease pathways, we observed a stronger inhibition than stimulation of inflammation (Supplementary Figure S6). Inhibition of the sirtuin pathway, combined with activation of the Hepatic Fibrosis signalling pathway is indication of potential first steps towards liver disease in the mid second trimester smoke exposed male foetuses since serotonin deficiency is considered to aggravate non-alcoholic fatty liver disease (NAFLD).^74^ Recently foetal growth, in particular femur length, in a longitudinal study was reported to be detectably slowed, if the mother smoked during pregnancy, as early at 16–20 weeks,^75^ while no body weight/length or organ weight associations with maternal smoking during pregnancy were observed. The authors concluded that organs like the brain and liver were protected at the expense of skeletal growth. Taken together, what we are observing in the foetal livers of foetuses whose mother smoke may be a combination of both protective and maladaptive responses, some leading to metabolic and liver disease. This is further reinforced by findings in two recent studies, associating maternal smoking during pregnancy with NAFLD and metabolic dysfunction hepatic disease.^76^^,^^77^

Our GWAS was conducted in offspring exposed to maternal smoking during pregnancy. The rationale to use this population was the shared genetics with their mothers, which should carry half of the genetic effects observed here. Therefore, our heritability estimate of around 3–4% is likely too small and would be higher in women who reported maternal smoking during pregnancy themselves. Nevertheless, the low heritability suggests that the effects observed are mostly epigenetic which is supported by previous research in the field.^22^^,^^23^^,^^78^ The results from our GWAS and TWAS indicate the involvement of genes related to tobacco addiction, fatty acid metabolism, and neurodegeneration, which together with our DGE pathway enrichment analysis presents a possible genetic basis for our observed associations and informs which mechanisms of action need to be further investigated. Although some sex bias was also observed in these analyses, very few genetic associations with maternal smoking during pregnancy were identified which could in part be due to the low sample sizes of each subgroup (Table 2) combined with the stringent Bonferroni correction threshold. Additionally, we investigated the pattern of overlap between the foetal DGE and adult TWAS. Similarly to stronger effects observed for males in foetal livers only (Fig. 1), we note that males also had a substantially higher degree of overlap with the overall TWAS at every stage of pregnancy in the second trimester as compared to females and the pattern remained largely similar when restricting the TWAS analysis to liver tissue only further strengthening our notion of males being affected to a greater degree by the phenotype. In addition, with the applied *P*-value cut-offs, we would expect around five genes to overlap between the liver TWAS and DGE by chance. Here, we observed an excess of overlapping genes in the later stages of pregnancy in males and females, indicating that some of the observed associations in the DGE may be influenced by inherited genetic variants. Building on those results, the genes that do overlap should be examined in more detail to find out their precise function in the context of maternal smoking in utero and their genetic control.

Finally, we examined the survival likelihood over five years of follow-up for the UK Biobank individuals. We note mortality was similar between males and females when exposed to maternal smoking during pregnancy. Importantly, the mortality risk was further increased if individuals smoked themselves, suggesting an interaction between uterine and active tobacco smoke exposure.^69^ Interestingly, when looking at subgroups according to smoking status we observed a markedly decreased mortality rate due to maternal smoking during pregnancy for never smokers compared to ever smokers. This observation has important implications for efforts to reduce the impact of pregnancy maternal smoking in our society. Briefly, men born to women who smoked during pregnancy should be strongly advised to never start smoking. This could greatly reduce their increased mortality due to maternal smoking during pregnancy. Such interventions could realistically be included in current anti-smoking counselling curricula as well as other preventive efforts.

One strength of this study is that we combine genetic and transcriptomic data from prenatal and middle-aged groups. This increases our ability to obtain a comprehensive understanding of the life-long biological consequences of pregnancy maternal smoking for the individual. Although the UK Biobank cohort provides considerable statistical power due to its large sample size, caution is warranted in the interpretation of the results. Indeed, we present biological associations related to maternal smoking during pregnancy. However, one weakness of the UK Biobank study cohort is that maternal smoking during pregnancy status is based on self-reporting by the offspring (i.e., the participants) rather than the mothers themselves. Additionally, variables such as paternal smoking for both UK Biobank and human foetal collection cohorts, as well as social deprivation index (human foetal collection only), were not available and consequently not accounted for. While we were not able to control directly for paternal smoking, all the foetuses included in the human foetal collection study had levels of cotinine that exceeded the smoking cut-off, in addition to mothers reporting smoking status during pregnancy. Consequently, the principal exposure to smoking was via the mother. Furthermore, despite not being able to control for deprivation index in the human foetal collection cohort, our current foetal collection of nearly 400 foetuses, found no significant association between maternal smoking and the Scottish Index of Multiple Deprivation.^79^ This could have potentially skewed and introduced bias in the observed associations regardless of the steps taken to remedy that. Moreover, the UK Biobank and the human foetal liver analyses use two different tissues (blood and liver, respectively) which probably explains why we did not observe all the results from foetal tissue mirrored in adult individuals. Finally, we note that many of the observed associations from our cox proportional hazards models have confidence intervals overlapping between the various strata. Thus, we do not have enough power to detect whether there is a statically significant difference in the mortality estimates between smoking and non-smoking males and only note that the effect is largely attuned in nonsmokers.

We observed that, compared to females, male foetuses are more affected by maternal smoking during pregnancy suggesting an early disruption and/or adaptation in developmental pathways. However, those effects were largely attenuated in middle-aged individuals, and we only observed a moderate sex difference in risk factors, future disease risk and mortality rates between men and women. Importantly, our results highlight that the increased mortality due to maternal smoking during pregnancy was severely attenuated in men who did not smoke themselves, as opposed to females for which hazard ratios were similar regardless of smoking status. Consequently, this imposes important implications for efforts to reduce the societal impact of maternal smoking during pregnancy. In our genetic dissection of pregnancy maternal smoking, we found that it is only weakly heritable in both offspring sexes. Nevertheless, we were able to highlight several genes and pathways likely to be involved in maternal smoking during pregnancy. By detailing those effects on the developing and post-natal liver, we have paved the way for future studies on the inherited molecular basis of maternal smoking during pregnancy.

## Contributors

Conception and study design: Felix Grassmann, Paul A. Fowler, Sophie Shaw and conception and design of foetal RNAseq study: Paul A. Fowler, Peter J. O'Shaughnessy, David C. Hay, John P. Iredale, Alex Douglas. Collection and assembly of data: Sophie Shaw, Alex Douglas. Data analysis and interpretation: Mihail Mihov, Felix Grassmann, Paul A. Fowler, Hannah Shoctor. Manuscript writing: Mihail Mihov, Felix Grassmann, Paul A. Fowler, Peter J. O'Shaughnessy, David C. Hay, John P. Iredale, Alex Douglas, Sophie Shaw, Hannah Shoctor. Final approval of manuscript: all authors. Access and verification of underlying data: Felix Grassmann and Paul A. Fowler.

## Data sharing statement

Access to the phenotypes and genotypes of the UK Biobank participants is governed by an application process at https://www.ukbiobank.ac.uk/. The code for the computation of the derived phenotypes, quality control, and all analyses will be hosted on GitHub at https://github.com/GrassmannLab. The summary statistics of the GWAS will be submitted to the GWAS catalogue https://www.ebi.ac.uk/gwas/.

## Declaration of interests

David C. Hay is a shareholder and director at Stimuliver ApS (Denmark) and Stemnovate Limited (UK). The remaining authors declare no competing interests for this manuscript.

## Appendix ASupplementary data

Supplementary Table S1 Baseline characteristics (mean ± S.D.) of the 80 human foetuses used for liver RNAseq, separated by sex and maternal smoking status. Other than cigarettes/day, no values were significantly different between groups (Tukey HSD or Wilcoxon tests as appropriate).

Supplementary Table S4

Supplementary Figure S1 **Top 20 canonical pathways associated with maternal smoking in the late gestational window male foetal livers.** Associations were picked based on z-score, p-value and at least 2-fold activation (positive z-score, 12 pathways) or inhibition (negative z-score, 2 pathways). A preponderance of activation across key pathways was observed. Three of the 20 pathways had z-scores of zero and 3 had no available activity pattern.

Supplementary Figure S2 **Quantile-quantile plot of the observed versus expected (theoretical) GWAS values for (a) overall cohort, (b) females, (c) males, (d) non-smokers, (e) smokers.** Red line represents the expected (theoretical) distribution of *P*-values. Common alleles are usually non-significant and follow the line whereas rare significant alleles tend to deviate from the expectation.

Supplementary Figure S3 **Summary of the genome-wide association studies of maternal smoking for females (a), males (b), smokers (c), and non-smokers (d).** Manhattan plots of (a) 4,954,542; (b) 4,925,554; (c) 5,374,679; and (d) 4,864,215 short nucleotide polymorphisms with minor allele frequency of at least 1%. Only genes harbouring exonic variants have been labelled. Red dots indicate genes in each region harbouring a statistically significant variant which has a CADD score greater than 12.37, indicating it is likely influencing that gene's function. The red line denotes genome-wide significance (*P* < 5 × 10^–8^, logistic regression). The estimated genomic inflation factors λ were (a) 1.14, (b) 1.135, (c) 1.109, (d) 1.152.

Supplementary Figure S4 **Summary of the transcriptome-wide association studies of maternal smoking for (a) females, (b) males, (c) smokers, (d) non-smokers.** Manhattan plots representing the association of GWAS variants with GTEx expression panels from 72 tissues. Only known coding genes reaching statistical significance are labelled. The red line denotes genome-wide significance (|Z-score| > 5.5). Direction of the triangles indicates genes predicted to be overexpressed (facing up) or underexpressed (facing down).

Supplementary Figure S5 **Upset plot representing the number of unique and overlapping genes between the differential gene expression in foetal livers from males and females at early (11–13), mid (14–16), late (17–19) weeks of gestation and overall (a) and liver specific (b) TWAS.** Set size represents the total number genes considered for each category. Intersection size represents the number of genes in the corresponding intersection. Connected dots in the intersection indicate overlap in DE and/or TWAS genes whereas solo dots indicate intersections with genes unique for the category. DE genes were considered at *P* < 0.05 or |Log Fold Change| > 0.5. TWAS genes were considered at *P* < 0.01 (logistic regression). Only intersections with a maximum of four overlapping category sets were considered.
