# Supplementary material for: Linking epidemiology and genomics of maternal smoking during pregnancy in utero and in ageing: a population-based study using human foetuses and the UK Biobank cohort
Source: eBioMedicine. 2025 Mar 12;114:105590. doi: 10.1016/j.ebiom.2025.105590 (PMC12121433; doi:10.1016/j.ebiom.2025.105590)
Supplement: Supplementary Table S1 — Baseline characteristics (mean ± S.D.) of the 80 human foetuses used for liver RNAseq, separated by sex and maternal smoking status. Other than cigarettes/day, no values were significantly different between groups (Tukey HSD or Wilcoxon tests as appropriate). [file mmc1.docx]

| **Variable** | **Female** | | **Male** | |
| --- | --- | --- | --- | --- |
|  | **Non-smoker** | **Smoker** | **Non-smoker** | **Smoker** |
| Number of samples (*n*) | 20 | 21 | 20 | 19 |
| Fetal age (weeks of gestation) | 14.5±2.2 | 14.8±2.3 | 15.0±2.2 | 14.8±2.2 |
| Fetal Body Weight (g) | 78.2±78.7 | 78.7±59.6 | 83.5±71.6 | 87.5±78.5 |
| Fetal crown-rump length (mm) | 97.8±28.2 | 97.2±22.5 | 101.9±25.9 | 103.7±32.3 |
| Maternal age (years) | 22.8±3.6 | 23.0±5.5 | 26.4±6.3 | 25.3±5.2 |
| Maternal BMI (kg/m^2^) | 24.6±4.5 | 25.2±5.5 | 24.9±4.9 | 25.0±6.1 |
| Cigarettes/day | 0 | 12.2±5.0 | 0 | 11.9±4.8 |

**S.D. – standard diviation*
