# Supplementary material for: Linking epidemiology and genomics of maternal smoking during pregnancy in utero and in ageing: a population-based study using human foetuses and the UK Biobank cohort
Source: eBioMedicine. 2025 Mar 12;114:105590. doi: 10.1016/j.ebiom.2025.105590 (PMC12121433; doi:10.1016/j.ebiom.2025.105590)
Supplement: Supplementary Table S2 [file mmc2.pdf]

**Supplementary Table 2.** 10 biological networks matching the DEGs associated with maternal smoking in livers from 17-19 weeks of gestation male fetuses and their top disease functions. Networks 5 and 8 overlap.

| ID | Molecules in Network | Score | Focus Molecules | Top Diseases and Functions |
|---|---|---|---|---|
| 1 | ACKR3,AGTR1,ARG2,ARRDC3,calpain,Collagen Alpha1,Creb,Cyclin E,EDNRB,Focal adhesion kinase,GJA1,Gpcr,GPR157,Hdac,HGF,histone deacetylase,HPSE,Hsp70,Hsp90 (family),Jnk,Ldh (complex),LDHA,N-cor,NBAS,NDRG1,Nos,ONECUT2,PARP,Pka,PKM,Raf,Rb,Relaxin,Rsk,SH3BP5 | 30 | 15 | [Cardiovascular System Development and Function, Cellular Development, Cellular Growth and Proliferation] |
| 2 | Alpha catenin,CG,CLDN5,Collagen type I (complex),collagen type i (family),CXCL8,DIO3,ERK,Fc gamma receptor,FSH,Hif1,Igm,Immunoglobulin,Integrin,Laminin (complex),Lh,LOXL2,Mapk,METRNL,MMP19,NHSL2,Notch,NPAS2,P2RX7,P38 MAPK,PDLIM3,PHLDA1,PI3K (family),PLC,PLOD2,Rap1,Tgf beta,TSH,VCL,Vegf | 25 | 13 | [Cell Death and Survival, Cell Morphology, Organismal Injury and Abnormalities] |
| 3 | AFAP1L2,APP,BCAR3,C11orf96,C3orf62,CMTM3,DDX39A,DGKI,DMAC2,EGLN3,ETAA1,F13A1,FAM229A,GBE1,GLCE,GPN2,GPR15,GTPase,HIF1A-AS3,IFFO1,MAFF,NENF,ODAM,PABIR3,PDK1,PLSCR1,SEC23A,SLFN13,SNX32,SYNE3,TBC1D30,TICRR,TMEM45B,ZC3HAV1L,ZCCHC14 | 25 | 13 | [Cell-To-Cell Signaling and Interaction, Cellular Compromise, Cellular Movement] |
| 4 | Activin (family),Akt,Alpha Actinin,Collagen type II,Collagen type III,Collagen type IV,Collagen(s),collagenase,Ctbp,elastase,Eotaxin,ETS,F11R,FAM13A,Fibrin,GP5,GYPB,H/K/NRAS,HIC1,IHH,Integrin alpha V beta 3,ITGB3,Mmp,PAPSS2,Pdgf (complex),PDGF-AA,Pkg,SEMA3D,SRGN,Stat3-Stat3,SVEP1,TCF,Tenascin,Thrombospondin,TTYH3 | 23 | 12 | [Dermatological Diseases and Conditions, Immunological Disease, Inflammatory Disease] |
| 5 | ADCY,ADRB,ANGPTL8,C1R,Cbp/p300,CD3 group,Cyclin D,cytochrome C,DGCR8,EFNA3,EGLN,EPO,ERK1/2,estrogen receptor,G protein,G protein alpha i,glutathione peroxidase,growth factor,Growth hormone,Hif,MORC3,Na ,K -ATPase,Osteocalcin,PFKP,Plc beta,Proinsulin,Rab5,RGS4,SAA,SCG5,SLC16A3,Sod,ST3GAL4,SYNE2,T3-TR-RXR | 23 | 12 | [Cell-To-Cell Signaling and Interaction, Nervous System Development and Function, Skeletal and Muscular System Development and Function] |
| 6 | ADAMTS4,Alp,c-Src,chemokine,collagen,ELF3,Fibrinogen,GPIIB-IIIA,HDL,Ifn,IL-1R,IL1,IL1R1,IL23,Integrin alpha 5 beta 1,KCNE4,LBP,NFkB (complex),NFkB (family),NfkB-RelA,NfkB1-RelA,Nr1h,OSMR,PDGF BB,Pro-inflammatory Cytokine,PTGIS,ROCK,SBNO2,STAT1/3/5 dimer,SYT7,THBS1,Tlr,Tnf (family),TNF receptor,TNFRSF11B | 20 | 11 | [Connective Tissue Disorders, Organismal Injury and Abnormalities, Skeletal and Muscular Disorders] |
| 7 | B4GALT1,BCL3,BCR (complex),C1q,cytokine,ETS2,FGF23,GOT,Gsk3,hemoglobin,ICAM1,IFN Beta,Iga,Ige,IgG,IgG1,IgG2a,Igg3,IL12 (complex),IL12 (family),Interferon alpha,JUNB,LDL,MHC Class II (complex),PELO,PI3K (complex),PLC gamma,RNA polymerase II,SAMSN1,SEMA4A,STAT3,STAT5a/b,STC2,SYK/ZAP,TCR | 20 | 11 | [Cell-mediated Immune Response, Cellular Development, Cellular Function and Maintenance] |
| 8 | 3',5'-cyclic-nucleotide phosphodiesterase,ALDOA,ARNT,beta-estradiol,CCDC17,CGB3 (includes others),Co2 ,cyclic AMP,cyclic GMP,DCT,EP300,ERK1/2,HTR7,ion channel,LINC01139,MIP1,MIR320,PCSK1N,PDE1/2/4,PDE4B,PDE5A,PDE6H,PDE9A,POMC,Pthr,RSF1,Sfk,SLC6A6,SMPDL3A,STAT,Sucrose Degradation V (Mammalian),TCEAL5,TPI1,UCN2,ZP4 | 14 | 8 | [Cell Signaling, Molecular Transport, Nucleic Acid Metabolism] |
| 9 | ADCYAP1R1,ADORA2A,AHSP,ARR3,caspase,CCBE1,CCL14,CCN6,CCR1,Ck2,DEFB123,EGFL6,GALR1,GPR132,Histone h3,Histone h4,Ifn gamma,LDLR,mir-185,MTA2,NPY5R,NTN5,PEPCK,PTGR1,RHPN2,SCRT1,SLC52A1,Smad2/3,SNHG5,SORL1,TBX1,TBX5,TIP60,trypsin,Ubiquitin | 12 | 7 | [Lymphoid Tissue Structure and Development, Organismal Development, Tissue Morphology] |
| 10 | 26s Proteasome,Actin,AMPK,Ap1,BAIAP2,Beta Arrestin,Calcineurin protein(s),Calmodulin,CD3,Cyclin A,F Actin,IKK (complex),Insulin,Integrin alpha L beta 2,JINK1/2,KANK1,MAP2K1/2,Mek,Mlc,MPZL2,MTORC1,Nfat (family),p70 S6k,p85 (pik3r),Pde4,PI3K p85,Pkc(s),Rac,RAS,Ras homolog,Shc,SOD2,Sos,SRC (family),Talin | 6 | 4 | [Auditory Disease, Hereditary Disorder, Neurological Disease] |

*Networks 5 and 8 Overlap*

https://qiagen.my.salesforce-sites.com/KnowledgeBase/articles/Basic_Technical_Q_A/Listing-of-Networks
