# Supplementary material for: Linking epidemiology and genomics of maternal smoking during pregnancy in utero and in ageing: a population-based study using human foetuses and the UK Biobank cohort
Source: eBioMedicine. 2025 Mar 12;114:105590. doi: 10.1016/j.ebiom.2025.105590 (PMC12121433; doi:10.1016/j.ebiom.2025.105590)
Supplement: Supplementary Table S3 [file mmc3.pdf]

**Supplementary Table 3.** Disease and biological functions pathways based on DEGs associated with maternal smoking in livers from 17-19 weeks of gestation male fetuses. The list is trimmed to only include those with p<0.05 and at least 2-fold decrease (23 functions) or increase (92 functions).

| Categories | Diseases or Functions Annotation | p-value | Predicted | Activation | Molecules | # Molecules |
|---|---|---|---|---|---|---|
| Inflammatory Response,Organismal Injury and Abnormalities | Inflammation of organ | 1.05E-11 | Decreased | -3.307 | ACKR3,ADORA2A,AGT | 38 |
| Cell Death and Survival,Organismal Injury and Abnormalities | Necrosis | 2.23E-11 | Decreased | -2.681 | ACKR3,ADORA2A,AGT | 53 |
| Organismal Injury and Abnormalities,Organismal Survival | Organismal death | 9.89E-11 | Decreased | -4.455 | ACKR3,ADAMTS4,ADC | 50 |
| Inflammatory Response,Organismal Injury and Abnormalities | Inflammation of body cavity | 7.86E-09 | Decreased | -2.999 | ACKR3,ADORA2A,AGT | 28 |
| Cell Death and Survival | Apoptosis | 1.31E-08 | Decreased | -3.155 | ACKR3,ADORA2A,AGT | 47 |
| Organismal Injury and Abnormalities,Renal and Urological Disease | Structural renal abnormality | 3.37E-08 | Decreased | -2.207 | ADAMTS4,AGTR1,CXC | 11 |
| Neurological Disease,Organismal Injury and Abnormalities | Degeneration of nervous system | 6.74E-07 | Decreased | -2.248 | ADORA2A,EDNRB,EP( | 13 |
| Neurological Disease,Organismal Injury and Abnormalities,Tissue Morphology | Neurodegeneration of nervous tissue | 1.05E-06 | Decreased | -2.249 | EDNRB,EPO,ETS2,HG | 12 |
| Cell Death and Survival | Cellular degradation | 1.6E-06 | Decreased | -2.249 | EDNRB,EPO,ETS2,HG | 12 |
| Neurological Disease,Organismal Injury and Abnormalities | Neurodegeneration | 1.74E-06 | Decreased | -2.239 | ADORA2A,EDNRB,EP( | 13 |
| Cell Death and Survival,Organismal Injury and Abnormalities | Cell death of tumor cell lines | 1.94E-06 | Decreased | -3.558 | ACKR3,ADORA2A,B4G | 32 |
| Cell Death and Survival,Organismal Injury and Abnormalities | Apoptosis of tumor cell lines | 2.48E-06 | Decreased | -2.806 | ACKR3,ADORA2A,B4G | 28 |
| Organ Morphology,Organismal Injury and Abnormalities,Renal and Urological Disease | Atrophy of kidney | 3.18E-06 | Decreased | -2.219 | ADAMTS4,AGTR1,EDN | 5 |
| Gastrointestinal Disease,Hepatic System Disease,Organismal Injury and Abnormalities | Liver lesion | 4.29E-06 | Decreased | -2.335 | ACKR3,ADAMTS4,ADC | 63 |
| Cell Death and Survival,Cellular Compromise,Neurological Disease,Organismal Injury and Abnormalities,Tissue Morphology | Degeneration of neurons | 5.52E-06 | Decreased | -2.066 | EDNRB,EPO,ETS2,HG | 11 |
| Cellular Compromise | Degeneration of cells | 7.15E-06 | Decreased | -2.579 | EDNRB,EPO,ETS2,HG | 12 |
| Organismal Injury and Abnormalities,Organismal Survival | Perinatal death | 8.25E-06 | Decreased | -2.447 | ACKR3,B4GALT1,CLDI | 15 |
| Developmental Disorder,Organismal Injury and Abnormalities | Hypoplasia of organ | 2.37E-05 | Decreased | -2.132 | B4GALT1,BCL3,EPO,F | 13 |
| Cell Death and Survival,Organismal Injury and Abnormalities | Cell death of carcinoma cell lines | 3.05E-05 | Decreased | -2.645 | ACKR3,BCL3,CXCL8,E | 13 |
| Inflammatory Disease,Inflammatory Response,Organismal Injury and Abnormalities,Skeletal and Muscular Disorders | Inflammation of muscle | 0.00004 | Decreased | -2 | ACKR3,HGF,IL1R1,ITG | 7 |
| Developmental Disorder,Organismal Injury and Abnormalities | Dysgenesis | 9.82E-05 | Decreased | -2.259 | AGTR1,B4GALT1,BCL | 14 |
| Cell Death and Survival,Organismal Injury and Abnormalities | Apoptosis of carcinoma cell lines | 0.000142 | Decreased | -2.279 | ACKR3,BCL3,ETS2,GJ | 11 |
| Organismal Injury and Abnormalities,Organismal Survival | Neonatal death | 0.000354 | Decreased | -2.003 | ACKR3,B4GALT1,CLDI | 10 |
| Cellular Movement | Cell movement | 1.58E-17 | Increased | 3.934 | ACKR3,ADORA2A,AGT | 60 |
| Cellular Movement | Migration of cells | 3.24E-15 | Increased | 3.135 | ACKR3,ADORA2A,AGT | 54 |
| Cellular Movement,Hematological System Development and Function,Immune Cell Trafficking | Cell movement of leukocytes | 1.35E-14 | Increased | 2.352 | ACKR3,ADORA2A,AGT | 32 |
| Cellular Movement,Immune Cell Trafficking | Leukocyte migration | 3.97E-14 | Increased | 2.771 | ACKR3,ADORA2A,AGT | 33 |
| Cellular Movement,Hematological System Development and Function,Immune Cell Trafficking,Inflammatory Response | Cell movement of phagocytes | 2.93E-13 | Increased | 2.453 | ADORA2A,B4GALT1,C | 26 |
| Cellular Movement | Cell movement of myeloid cells | 1.63E-12 | Increased | 2.329 | ADORA2A,B4GALT1,C | 25 |
| Cancer,Dermatological Diseases and Conditions,Organismal Injury and Abnormalities | Skin tumor | 1.22E-11 | Increased | 2.236 | ACKR3,ADAMTS4,ADC | 90 |
| Cellular Movement | Invasion of cells | 2.44E-10 | Increased | 2.014 | ACKR3,ALDOA,ARRD( | 34 |
| Cellular Function and Maintenance | Cellular homeostasis | 2.91E-10 | Increased | 2.056 | ADORA2A,AGTR1,ALD | 39 |
| Cancer,Organismal Injury and Abnormalities | Metastasis | 1.35E-09 | Increased | 2.072 | ACKR3,ADORA2A,C1F | 31 |
| Cancer,Organismal Injury and Abnormalities | Advanced malignant tumor | 1.81E-09 | Increased | 2.072 | ACKR3,ADORA2A,C1F | 32 |
| Cancer,Organismal Injury and Abnormalities | Invasive cancer | 1.9E-09 | Increased | 2.072 | ACKR3,ADAMTS4,ADC | 35 |
| Cell-To-Cell Signaling and Interaction | Binding of blood cells | 2.73E-09 | Increased | 3.006 | ADORA2A,B4GALT1,C | 17 |
| Cell-To-Cell Signaling and Interaction | Adhesion of blood cells | 3.21E-09 | Increased | 2.788 | ADORA2A,B4GALT1,C | 16 |
| Cellular Movement | Cell movement of tumor cell lines | 3.4E-09 | Increased | 2.603 | ACKR3,ALDOA,ARRD( | 33 |
| Cell-To-Cell Signaling and Interaction,Hematological System Development and Function | Binding of leukocytes | 4.13E-09 | Increased | 3.134 | ADORA2A,B4GALT1,C | 16 |
| Organismal Survival | Survival of organism | 5E-09 | Increased | 2.005 | ADORA2A,AGTR1,BCL | 25 |
| Cellular Movement | Mobilization of cells | 8.8E-09 | Increased | 2.007 | CCR1,CXCL8,EPO,F11 | 8 |
| Cell-To-Cell Signaling and Interaction,Hematological System Development and Function,Immune Cell Trafficking | Adhesion of immune cells | 1.03E-08 | Increased | 3.062 | ADORA2A,B4GALT1,C | 15 |
| Cellular Movement | Homing of cells | 1.29E-08 | Increased | 2.293 | ACKR3,ADORA2A,AGT | 19 |
| Cell Death and Survival | Cell survival | 1.31E-08 | Increased | 3.842 | ACKR3,ADORA2A,ALD | 35 |
| Cell Death and Survival | Cell viability | 1.56E-08 | Increased | 4.07 | ACKR3,ADORA2A,ALD | 34 |
| Cell-To-Cell Signaling and Interaction,Cellular Movement | Recruitment of cells | 2.63E-08 | Increased | 2.204 | B4GALT1,BCL3,CCR1, | 15 |
| Cellular Movement | Chemotaxis | 3.27E-08 | Increased | 2.142 | ACKR3,ADORA2A,AGT | 18 |
| Cell-To-Cell Signaling and Interaction | Adhesion of tumor cell lines | 4.46E-08 | Increased | 3.598 | B4GALT1,CXCL8,F11F | 14 |
| Cell-To-Cell Signaling and Interaction,Hematological System Development and Function,Immune Cell Trafficking,Inflammatory Response | Activation of phagocytes | 6.36E-08 | Increased | 2.335 | CXCL8,EPO,GJA1,HGI | 15 |
| Cellular Movement,Hematological System Development and Function,Immune Cell Trafficking,Inflammatory Response | Migration of phagocytes | 9.92E-08 | Increased | 2.225 | ADORA2A,CCR1,CXCL | 13 |
| Cell-To-Cell Signaling and Interaction,Hematological System Development and Function | Activation of myeloid cells | 1.32E-07 | Increased | 2.334 | CXCL8,EPO,GJA1,HPS | 14 |
| Cellular Movement,Skeletal and Muscular System Development and Function | Migration of smooth muscle cells | 1.38E-07 | Increased | 2.773 | ACKR3,CXCL8,F11R,G | 10 |
| Cell-To-Cell Signaling and Interaction,Cellular Movement,Hematological System Development and Function,Immune Cell Trafficking,Inflammatory Response | Recruitment of phagocytes | 1.65E-07 | Increased | 2.088 | B4GALT1,BCL3,CCR1, | 12 |
| Cell Morphology | Orientation of cells | 1.83E-07 | Increased | 2.804 | ADORA2A,CXCL8,ICAI | 11 |
| Cardiovascular System Development and Function,Cellular Movement | Migration of endothelial cells | 2.06E-07 | Increased | 2.378 | ACKR3,CCBE1,CXCL8 | 14 |

| | | | | | | |
|---|---|---|---|---|---|---|
| Cellular Development,Cellular Growth and Proliferation | Colony formation of cells | 2.14E-07 | Increased | 2.652 | ACKR3,CXCL8,DGCR8 | 18 |
| Cellular Movement | Migration of tumor cell lines | 2.45E-07 | Increased | 2.439 | ACKR3,ALDOA,ARRD | 28 |
| Cellular Development,Cellular Growth and Proliferation | Cell proliferation of tumor cell lines | 2.66E-07 | Increased | 2.59 | ACKR3,ADAMTS4,AGT | 39 |
| Cell Death and Survival | Cell viability of blood cells | 2.68E-07 | Increased | 2.484 | ACKR3,BCL3,CXCL8,D | 12 |
| Cell-To-Cell Signaling and Interaction | Interaction of tumor cell lines | 2.81E-07 | Increased | 3.772 | B4GALT1,CXCL8,F11F | 15 |
| Cell-To-Cell Signaling and Interaction,Cellular Movement,Hematological System Development and Function,Immune Cell Trafficking | Recruitment of leukocytes | 3.74E-07 | Increased | 2.345 | B4GALT1,BCL3,CCR1, | 13 |
| Molecular Transport | Transport of molecule | 8.34E-07 | Increased | 2.287 | ADORA2A,B4GALT1,B | 30 |
| Cellular Development,Cellular Growth and Proliferation,Hematological System Development and Function,Hematopoiesis,Lymphoid Tissue Structure and Development,Tissue Development | Leukopoiesis | 8.38E-07 | Increased | 2.244 | BCL3,CXCL8,DGCR8,E | 21 |
| Cell-To-Cell Signaling and Interaction,Hematological System Development and Function,Immune Cell Trafficking | Binding of granulocytes | 1.19E-06 | Increased | 2.156 | ADORA2A,B4GALT1,C | 8 |
| Inflammatory Disease,Organismal Injury and Abnormalities,Respiratory Disease | Fibrosis of lung | 1.67E-06 | Increased | 2.177 | ADORA2A,AGTR1,ARC | 14 |
| Organismal Injury and Abnormalities | Pulmonary fibrosis or aplastic anemia | 1.69E-06 | Increased | 2.177 | ADORA2A,AGTR1,ARC | 15 |
| Cancer | Sphere formation of tumor cell lines | 2.2E-06 | Increased | 2.44 | ACKR3,CXCL8,EGLN3 | 8 |
| Carbohydrate Metabolism | Glycolysis of cells | 2.44E-06 | Increased | 2.689 | ALDOA,ITGB3,LDHA,P | 10 |
| Cell-To-Cell Signaling and Interaction,Cellular Assembly and Organization | Cell-cell contact | 2.59E-06 | Increased | 2.798 | ADORA2A,B4GALT1,C | 20 |
| Cancer | Sphere formation of carcinoma cell lines | 3.85E-06 | Increased | 2.201 | CXCL8,ICAM1,PDE4B, | 5 |
| Cellular Development,Cellular Growth and Proliferation,Connective Tissue Development and Function,Skeletal and Muscular System Development and Function,Tissue Development | Proliferation of osteoblasts | 4.26E-06 | Increased | 2.423 | EPO,HGF,HPSE,ITGB | 6 |
| Cellular Function and Maintenance | Homeostasis of leukocytes | 4.41E-06 | Increased | 2.034 | BCL3,ELF3,ETS2,HGF | 16 |
| Carbohydrate Metabolism,Cellular Function and Maintenance | Glycolysis of tumor cell lines | 4.74E-06 | Increased | 2.379 | ALDOA,ITGB3,LDHA,P | 7 |
| Inflammatory Response,Organismal Injury and Abnormalities | Acute inflammation | 5.54E-06 | Increased | 2.213 | B4GALT1,EDNRB,HPS | 5 |
| Inflammatory Disease,Inflammatory Response,Organismal Injury and Abnormalities,Respiratory Disease | Inflammation of airway | 5.96E-06 | Increased | 2.449 | ADORA2A,AGTR1,ALD | 12 |
| Cardiovascular System Development and Function,Organismal Development | Formation of blood vessel | 7.17E-06 | Increased | 2.433 | DGCR8,EPO,HGF,ICAI | 8 |
| Cell Morphology | Polarization of cells | 9.15E-06 | Increased | 2.433 | ADORA2A,CXCL8,ICAI | 9 |
| Cell-mediated Immune Response,Cellular Development,Cellular Function and Maintenance,Cellular Growth and Proliferation,Embryonic Development,Hematological System Development and Function,Hematopoiesis,Lymphoid Tissue Structure and Development,Organ Development,Organismal Development,Tissue Development | Differentiation of helper T lymphocytes | 9.4E-06 | Increased | 2.404 | BCL3,ELF3,ICAM1,IL1 | 9 |
| Cell Morphology,Cellular Movement | Cell spreading | 0.00001 | Increased | 2.533 | BAIAP2,CXCL8,EDNRE | 10 |
| Cell-To-Cell Signaling and Interaction | Adhesion of colorectal cancer cell lines | 1.06E-05 | Increased | 2.19 | HGF,ICAM1,PHLDA1,P | 5 |
| Cellular Function and Maintenance | Lymphocyte homeostasis | 1.49E-05 | Increased | 2.034 | BCL3,ELF3,ETS2,HGF | 15 |
| Cell-mediated Immune Response,Cellular Development,Cellular Function and Maintenance,Cellular Growth and Proliferation,Embryonic Development,Hematological System Development and Function,Hematopoiesis,Lymphoid Tissue Structure and Development,Organ Development,Organismal Development,Tissue Development | Differentiation of T lymphocytes | 2.21E-05 | Increased | 2.594 | BCL3,ELF3,ICAM1,IHH | 12 |
| Tissue Development | Tubulation of epithelial tissue | 2.39E-05 | Increased | 2.391 | CXCL8,EDNRB,EPO,F | 7 |
| Cellular Growth and Proliferation | Outgrowth of cells | 2.39E-05 | Increased | 2.694 | ADORA2A,BAIAP2,GJ | 12 |
| Cell-mediated Immune Response,Cellular Development,Cellular Function and Maintenance,Cellular Growth and Proliferation,Embryonic Development,Hematological System Development and Function,Hematopoiesis,Lymphoid Tissue Structure and Development,Organ Development,Organismal Development,Tissue Development | T cell development | 2.75E-05 | Increased | 2.611 | BCL3,ELF3,ETS2,ICAN | 14 |
| Cardiovascular System Development and Function,Cell Morphology,Cellular Development,Organismal Development,Tissue Development | Tubulation of vascular endothelial cells | 2.81E-05 | Increased | 2.396 | CXCL8,EDNRB,EPO,F | 6 |
| Cellular Growth and Proliferation,Tissue Development | Proliferation of neural cells | 3.36E-05 | Increased | 2.822 | ADORA2A,BAIAP2,CCI | 16 |
| Cell Death and Survival,Hematological System Development and Function | Cell viability of myeloid cells | 3.61E-05 | Increased | 2.367 | CXCL8,EPO,HGF,ICAN | 6 |
| Cellular Development,Cellular Growth and Proliferation,Nervous System Development and Function,Tissue Development | Outgrowth of neurons | 0.000041 | Increased | 2.276 | ADORA2A,BAIAP2,GJ | 11 |
| Cell Death and Survival | Cell viability of tumor cell lines | 4.33E-05 | Increased | 3.246 | ACKR3,ADORA2A,ALD | 21 |
| Organismal Development | Size of body | 6.37E-05 | Increased | 2.02 | AGTR1,B4GALT1,DIO | 17 |
| Lipid Metabolism,Small Molecule Biochemistry | Fatty acid metabolism | 7.03E-05 | Increased | 2.137 | ADORA2A,B4GALT1,C | 14 |
| Cancer,Organismal Injury and Abnormalities,Tumor Morphology | Invasion of tumor | 7.54E-05 | Increased | 2.425 | ACKR3,DGCR8,HGF,H | 8 |
| Cellular Development,Cellular Growth and Proliferation | Development of carcinoma cell lines | 7.67E-05 | Increased | 2.219 | DGCR8,HGF,HPSE,PK | 6 |
| Cancer,Cellular Movement,Organismal Injury and Abnormalities,Tumor Morphology | Invasion of tumor cells | 7.77E-05 | Increased | 2.425 | ACKR3,HGF,HPSE,ICA | 7 |
| Cell Morphology,Tissue Development | Tubulation of cells | 7.98E-05 | Increased | 2.391 | CXCL8,EDNRB,EPO,F | 7 |
| Cell Morphology | Contraction of cells | 8.22E-05 | Increased | 2.219 | AGTR1,CXCL8,EDNRB | 6 |
| Cellular Development,Cellular Growth and Proliferation,Nervous System Development and Function,Tissue Development | Proliferation of neuronal cells | 8.51E-05 | Increased | 2.667 | ADORA2A,BAIAP2,EP | 13 |
| Cellular Development | Epithelial-mesenchymal transition | 8.86E-05 | Increased | 2.017 | CXCL8,HGF,ITGB3,LD | 10 |
| Cellular Movement,Hair and Skin Development and Function | Migration of keratinocytes | 8.98E-05 | Increased | 2.173 | ALDOA,CXCL8,HGF,IC | 5 |
| Cellular Development,Cellular Growth and Proliferation,Embryonic Development,Hematological System Development and Function,Hematopoiesis,Lymphoid Tissue Structure and Development,Organ Development,Organismal Development,Tissue Development | Lymphopoiesis | 9.29E-05 | Increased | 2.04 | BCL3,ELF3,EPO,ETS2 | 15 |
| Cellular Development,Cellular Growth and Proliferation,Nervous System Development and Function,Organismal Development,Tissue Development | Development of neurons | 9.69E-05 | Increased | 2.499 | ADAMTS4,ADORA2A,E | 19 |

| | | | | | | |
|---|---|---|---|---|---|---|
| Cellular Development | Epithelial-mesenchymal transition of tumor cell lines | 0.000107 | Increased | 2.002 | CXCL8,HGF,ITGB3,LD | 8 |
| Cell Morphology,Cellular Development | Branching of cells | 0.000113 | Increased | 2.199 | ADAMTS4,BAIAP2,EF | 12 |
| Cell Death and Survival | Cell viability of lung cancer cell lines | 0.000134 | Increased | 2.051 | HGF,PDK1,PHLDA1,P | 7 |
| Cell Signaling,Cellular Function and Maintenance,Molecular Transport,Vitamin and Mineral Metabolism | Flux of Ca2+ | 0.000162 | Increased | 2.076 | CCR1,CXCL8,EPO,ET | 9 |
| Cellular Development,Cellular Growth and Proliferation,Nervous System Development and Function,Tissue Development | Outgrowth of neurites | 0.000167 | Increased | 2.096 | ADORA2A,BAIAP2,GJ | 10 |
| Nucleic Acid Metabolism,Small Molecule Biochemistry | Synthesis of nucleotide | 0.000167 | Increased | 2.491 | ADORA2A,AGTR1,AL | 10 |
| Cell Death and Survival | Regeneration of cells | 0.000182 | Increased | 2.414 | EPO,GJA1,HGF,ICAM1 | 6 |
| Cell Morphology | Sprouting | 0.000189 | Increased | 2.01 | ADAMTS4,BAIAP2,EF | 12 |
| Energy Production,Nucleic Acid Metabolism,Small Molecule Biochemistry | Synthesis of ATP | 0.000199 | Increased | 2.132 | ALDOA,GJA1,PKM,PT | 6 |
| Cell Cycle,Gene Expression | Binding of protein binding site | 0.000207 | Increased | 2.095 | CXCL8,EPO,ETS2,HG | 8 |
| Nucleic Acid Metabolism,Small Molecule Biochemistry | Metabolism of nucleotide | 0.000241 | Increased | 2.491 | ADORA2A,AGTR1,AL | 11 |
| Cellular Development,Cellular Growth and Proliferation,Nervous System Development and Function,Tissue Development | Growth of neurites | 0.000257 | Increased | 2.321 | ADORA2A,BAIAP2,GJ | 11 |
| Cell Death and Survival | Survival of neural cells | 0.000272 | Increased | 2.385 | ADORA2A,CCR1,EFNA | 8 |
| Cell Signaling,Cellular Function and Maintenance,Molecular Transport,Vitamin and Mineral Metabolism | Influx of Ca2+ | 0.000323 | Increased | 2.183 | CCR1,EPO,GJA1,JUN | 7 |
| Nucleic Acid Metabolism,Small Molecule Biochemistry | Synthesis of purine nucleotide | 0.00035 | Increased | 2.132 | AGTR1,ALDOA,GJA1, | 7 |

https://qiagen.my.salesforce-sites.com/KnowledgeBase/articles/Basic_Technical_Q_A/Accessing-and-Using-Functional-Analysis
