# Supplementary material for: Linking epidemiology and genomics of maternal smoking during pregnancy in utero and in ageing: a population-based study using human foetuses and the UK Biobank cohort
Source: eBioMedicine. 2025 Mar 12;114:105590. doi: 10.1016/j.ebiom.2025.105590 (PMC12121433; doi:10.1016/j.ebiom.2025.105590)
Supplement: Supplementary Table S4 [file mmc4.pdf]

**Supplementary Table 4.** Regulator effects. The algorithm connects upstream regulators, dataset molecules and downstream functions or diseases affected in DEGs associated with maternal smoking in livers from 17-19 weeks of gestation male fetuses.

| ID | Consistency Score | Node Total | Regulator Total | Regulators | Target Total | Target Molecules in Dataset | Disease & Function | Diseases & Functions | Known Regulator-Disease/Function Relationship |
|---|---|---|---|---|---|---|---|---|---|
| 1 | 105.46 | 104 | 34 | ARNT,CD44,CD5,CEBPB,COLLAGENASE (family),CYTOKINE (family),ECSIT,ERK1/2 (family),ESRRG,FLCN,FN1,GPR174,HIF1 (complex),HSPB8,IKBKE,IL22,IL3,IL6R,JAK1,KLF6,LEP,let-7 (includes others),let-7a-5p (and other miRNAs w/seed GAGGUAG),LINC01139,miR-124-3p (and other miRNAs w/seed AAGGCAC),OSM,PALMD,PDGFB,PIK3R1,SLC2A3,TCR (complex),TFAM,TNFRSF1A,YBX1 | 47 | ACKR3,ADAMTS4,ADORA2A,AGTR1,ALDOA,B4GALT1,BAIAP2,BCL3,C1R,CCR1,CXCL8,EPO,ETS2,F11R,FGF23,GJA1,GP5,HGF,ICAM1,IL1R1,ITGB3,JUNB,KANK1,LBP,LDHA,LDLR,LOXL2,METRNL,MMP19,MPZL2,OSMR,P2RX7,PAPSS2,PDE4B,PDK1,PFKP,PHLDA1,PKM,PLOD2,RGS4,SBNO2,SLC16A3,SOD2,STAT3,THBS1,TNFRSF11B,VCL | 23 | Adhesion of blood cells,Adhesion of tumor cell lines,Advanced malignant tumor,Apoptosis,Binding of leukocytes,Cell death of carcinoma cell lines,Cell proliferation of tumor cell lines,Cell spreading,Cell viability of myeloid cells,Cell viability of tumor cell lines,Cell-cell contact,Chemotaxis,Colony formation of cells,Differentiation of helper T lymphocytes,Glycolysis of tumor cell lines,Invasion of cells,Invasive cancer,Migration of endothelial cells,Migration of keratinocytes,Migration of phagocytes,Migration of tumor cell lines,Organismal death,Sphere formation of tumor cell lines | 36% (279/782) |
| 2 | 95.833 | 98 | 28 | ARNT,CD5,CEBPB,COLLAGENASE (family),CYTOKINE (family),ECSIT,ERK1/2 (family),F2,FLCN,GPR174,HIF1 (complex),HSPB8,IL15,IL22,IL3,JAK1,LINC01139,MYD88,NFKB (complex),NQO1,OSM,PIK3R1,PRKCD,RELA,SLC2A3,TNFRSF1A,TNFRSF1B,YBX1 | 47 | ACKR3,ADAMTS4,ADORA2A,AGTR1,ALDOA,B4GALT1,BAIAP2,BCL3,C1R,CCR1,CXCL8,DIO3,EDNRB,ELF3,EPO,ETS2,FGF23,GJA1,GP5,HGF,ICAM1,IL1R1,ITGB3,JUNB,LBP,LDHA,LDLR,LOXL2,MAFF,MPZL2,OSMR,P2RX7,PDE4B,PDK1,PFKP,PHLDA1,PKM,PLSCR1,RGS4,SBNO2,SLC16A3,SOD2,SORL1,SRGN,STAT3,THBS1,TNFRSF11B | 23 | Adhesion of blood cells,Adhesion of tumor cell lines,Advanced malignant tumor,Apoptosis,Binding of leukocytes,Cell death of tumor cell lines,Cell movement of myeloid cells,Cell proliferation of tumor cell lines,Cell spreading,Cell viability,Cell-cell contact,Chemotaxis,Colony formation of cells,Differentiation of helper T lymphocytes,Glycolysis of tumor cell lines,Invasion of cells,Invasive cancer,Migration of endothelial cells,Migration of keratinocytes,Migration of phagocytes,Migration of tumor cell lines,Organismal death,Sphere formation of tumor cell lines | 41% (262/644) |
| 3 | 79.158 | 80 | 20 | AHR,CCL5,CSF1,CSF2,CYTOKINE (family),IKBKB,IL1A,IL22,IL6,IL6R,KITLG,KLF6,LACTB,LEP,miR-34a-5p (and other miRNAs w/seed GGCAGUG),PDGF-BB (complex),PDGFB,SP1,TCR (complex),TLR4 | 42 | ACKR3,ADAMTS4,ADORA2A,AGTR1,ALDOA,ARG2,BCL3,CCR1,CXCL8,EDNRB,EGLN3,EPO,ETS2,FGF23,GJA1,HGF,HPSE,ICAM1,IL1R1,ITGB3,JUNB,LDHA,LDLR,LOXL2,MMP19,NDRG1,OSMR,P2RX7,PDE4B,PDK1,PHLDA1,PKM,PLSCR1,SAMSN1,SEMA4A,SLC6A6,SOD2,SORL1,STAT3,THBS1,TNFRSF11B,VCL | 18 | Apoptosis,Cell death of carcinoma cell lines,Cell viability,Development of neurons,Differentiation of T lymphocytes,Dysgenesis,Epithelial-mesenchymal transition,Interaction of tumor cell lines,Migration of smooth muscle cells,Migration of tumor cell lines,Outgrowth of cells,Polarization of cells,Proliferation of osteoblasts,Sphere formation of tumor cell lines,Survival of neural cells,Synthesis of nucleotide,Tubulation of cells,Tubulation of epithelial tissue | 39% (142/360) |
| 4 | 75.744 | 77 | 20 | CCL5,CD5,COLLAGENASE (family),CSF2,CYTOKINE (family),EGLN (family),HIF1 (complex),IKBKB,IL15,IL1A,IL22,IL3,IL6,IL6R,KLF6,LEP,LIF,NQO1,PIK3R1,TCR (complex) | 41 | ADAMTS4,ADORA2A,AGTR1,ALDOA,BCL3,CCR1,CXCL8,DIO3,EFNA3,EGLN3,ELF3,EPO,ETS2,FGF23,HGF,HPSE,ICAM1,IL1R1,ITGB3,JUNB,LDHA,LDLR,LOXL2,MMP19,OSMR,P2RX7,PDE4B,PDK1,PFKP,PHLDA1,PKM,RGS4,SEMA4A,SLC16A3,SOD2,SORL1,SRGN,STAT3,THBS1,TNFRSF11B,TPI1 | 16 | Adhesion of tumor cell lines,Advanced malignant tumor,Apoptosis,Cell death of tumor cell lines,Cell proliferation of tumor cell lines,Cell viability,Differentiation of T lymphocytes,Epithelial-mesenchymal transition,Glycolysis of cells,Hypoplasia of organ,Invasion of cells,Invasive cancer,Migration of smooth muscle cells,Migration of tumor cell lines,Proliferation of osteoblasts,Synthesis of ATP | 44% (141/320) |
| 5 | 75.262 | 87 | 21 | CD5,CEBPB,COLLAGENASE (family),CSF2,ECSIT,ERK1/2 (family),HIF1 (complex),HSPB8,IKBKB,IL22,IL3,IL5,JAK1,KLF6,LIF,LINC01139,NFKB (complex),PIK3R1,SLC2A3,TNFRSF1A,YBX1 | 40 | ACKR3,ALDOA,B4GALT1,BCL3,C1R,CCR1,CXCL8,EDNRB,EGLN3,ELF3,EPO,ETS2,GJA1,GP5,HGF,HPSE,ICAM1,IL1R1,ITGB3,JUNB,LBP,LDHA,LDLR,LOXL2,NDRG1,OSMR,P2RX7,PDE4B,PDK1,PFKP,PHLDA1,PKM,RGS4,SEMA4A,SLC16A3,SOD2,SORL1,STAT3,THBS1,TNFRSF11B | 26 | Adhesion of blood cells,Adhesion of tumor cell lines,Advanced malignant tumor,Apoptosis,Binding of leukocytes,Cell death of tumor cell lines,Cell proliferation of tumor cell lines,Cell spreading,Cell viability,Cell-cell contact,Cellular degradation,Chemotaxis,Colony formation of cells,Degeneration of cells,Degeneration of nervous system,Differentiation of helper T lymphocytes,Glycolysis of tumor cell lines,Invasion of cells,Invasive cancer,Migration of endothelial cells,Migration of keratinocytes,Migration of phagocytes,Migration of tumor cell lines,Neurodegeneration,Sphere formation of tumor cell lines,Synthesis of nucleotide | 35% (189/546) |
| 6 | 70.362 | 100 | 30 | ARNT,BCR (complex),CD5,CEBPB,COLLAGENASE (family),ECSIT,EGLN2,ERK1/2 (family),F2,FLCN,HIF1 (complex),HSPB8,IL22,IL3,JAK1,let-7 (includes others),LIF,LINC01139,MYCN,NFKB (complex),NPM1,OSCAR,PIK3R1,PSME3,RELA,SLC2A3,SMAD3,TNFRSF1A,TNFRSF1B,YBX1 | 42 | ACKR3,ALDOA,B4GALT1,BCL3,C1R,CCR1,CXCL8,DIO3,EDNRB,EGLN3,ELF3,EPO,FGF23,GJA1,GP5,HGF,HPSE,ICAM1,IL1R1,ITGB3,JUNB,LBP,LDHA,LDLR,LOXL2,MMP19,NDRG1,OSMR,P2RX7,PDE4B,PDK1,PFKP,PKM,PLOD2,RGS4,SAMSN1,SLC16A3,SOD2,SRGN,STAT3,THBS1,TNFRSF11B | 28 | Adhesion of blood cells,Advanced malignant tumor,Apoptosis,Binding of leukocytes,Cell death of tumor cell lines,Cell movement of myeloid cells,Cell movement of tumor cell lines,Cell proliferation of tumor cell lines,Cell spreading,Cell viability,Cell-cell contact,Cellular degradation,Chemotaxis,Colony formation of cells,Degeneration of cells,Degeneration of nervous system,Differentiation of helper T lymphocytes,Fatty acid metabolism,Glycolysis of tumor cell lines,Interaction of tumor cell lines,Invasion of cells,Invasive cancer,Migration of endothelial cells,Migration of keratinocytes,Migration of phagocytes,Neurodegeneration,Organismal death,Sphere formation of tumor cell lines | 35% (298/840) |
| 7 | 69.653 | 75 | 17 | CCL5,CYTOKINE (family),EGR1,ERK (family),IKBKB,IL1A,IL6,IL6R,KITLG,KLF6,LEP,P38 MAPK (family),PDGF-BB (complex),PDGFB,SP1,TCR (complex),TLR4 | 41 | ACKR3,ADAMTS4,ADORA2A,AGTR1,ALDOA,BAIAP2,BCL3,CCR1,CXCL8,DIO3,EDNRB,EGLN3,EPO,ETS2,FGF23,GJA1,HGF,HPSE,ICAM1,IL1R1,ITGB3,JUNB,LBP,LDHA,LDLR,MAFF,MMP19,NDRG1,P2RX7,PDE4B,PDK1,PHLDA1,PKM,PLSCR1,RGS4,SAMSN1,SEMA4A,SOD2,STAT3,THBS1,TNFRSF11B | 17 | Binding of granulocytes,Cell viability,Development of neurons,Differentiation of T lymphocytes,Dysgenesis,Epithelial-mesenchymal transition,Interaction of tumor cell lines,Migration of smooth muscle cells,Migration of tumor cell lines,Outgrowth of cells,Polarization of cells,Proliferation of osteoblasts,Sphere formation of tumor cell lines,Survival of neural cells,Synthesis of nucleotide,Tubulation of cells,Tubulation of epithelial tissue | 43% (125/289) |
| 8 | 69.445 | 85 | 20 | APC,CCL5,CYTOKINE (family),FLCN,IKBKB,IL1A,IL6,IL6R,KITLG,KLF6,LACTB,LEP,let-7 (includes others),miR-34a-5p (and other miRNAs w/seed GGCAGUG),NFKB (complex),PDGF-BB (complex),PDGFB,SP1,TCR (complex),TLR4 | 46 | ACKR3,ADAMTS4,ADORA2A,AGTR1,ALDOA,ARG2,B4GALT1,BCL3,CCR1,CXCL8,EDNRB,EGLN3,ELF3,EPO,ETS2,F13A1,FGF23,GJA1,GP5,HGF,HPSE,ICAM1,IL1R1,ITGB3,JUNB,LBP,LDHA,LDLR,LOXL2,MMP19,NDRG1,OSMR,P2RX7,PDE4B,PDK1,PHLDA1,PKM,PLSCR1,SAMSN1,SBNO2,SEMA4A,SLC6A6,SOD2,STAT3,THBS1,TNFRSF11B | 19 | Adhesion of blood cells,Apoptosis,Cell viability,Development of neurons,Differentiation of T lymphocytes,Dysgenesis,Epithelial-mesenchymal transition,Interaction of tumor cell lines,Invasion of cells,Migration of smooth muscle cells,Migration of tumor cell lines,Organismal death,Outgrowth of cells,Polarization of cells,Sphere formation of tumor cell lines,Survival of neural cells,Synthesis of nucleotide,Tubulation of cells,Tubulation of epithelial tissue | 46% (173/380) |
| 9 | 68.647 | 52 | 10 | Brd4,CD44,HSPB8,LINC01139,OSCAR,PIK3R1,SLC2A3,TNFRSF1A,TNFRSF1B,YBX1 | 20 | ACKR3,ALDOA,C1R,CXCL8,EDNRB,HGF,ICAM1,IL1R1,LBP,LDHA,LOXL2,MMP19,P2RX7,PDK1,PFKP,PKM,SLC16A3,SOD2,THBS1,TPI1 | 22 | Activation of myeloid cells,Activation of phagocytes,Adhesion of blood cells,Adhesion of tumor cell lines,Advanced malignant tumor,Binding of leukocytes,Cell movement of myeloid cells,Cell movement of phagocytes,Cell proliferation of tumor cell lines,Cell spreading,Cell viability,Cell-cell contact,Chemotaxis,Fatty acid metabolism,Glycolysis of tumor cell lines,Inflammation of airway,Invasion of cells,Invasive cancer,Migration of endothelial cells,Migration of keratinocytes,Migration of tumor cell lines,Recruitment of phagocytes | 29% (63/220) |
| 10 | 54.271 | 62 | 13 | BCR (complex),CD5,CEBPB,COLLAGENASE (family),ERK1/2 (family),HSPB8,IL22,LIF,MMP9,PIK3R1,RELA,TNFRSF1A,YBX1 | 27 | ALDOA,B4GALT1,BCL3,CCR1,CLDN5,CXCL8,ELF3,GJA1,HGF,HPSE,ICAM1,IL1R1,ITGB3,JUNB,LBP,LDHA,LDLR,P2RX7,PDE4B,PDK1,PKM,RGS4,SAMSN1,SOD2,STAT3,THBS1,TNFRSF11B | 22 | Adhesion of blood cells,Adhesion of tumor cell lines,Advanced malignant tumor,Apoptosis,Binding of leukocytes,Cell death of tumor cell lines,Cell movement of myeloid cells,Cell spreading,Cell viability,Cell-cell contact,Cellular degradation,Chemotaxis,Degeneration of cells,Degeneration of nervous system,Differentiation of helper T lymphocytes,Invasion of cells,Invasive cancer,Migration of endothelial cells,Migration of keratinocytes,Migration of phagocytes,Migration of tumor cell lines,Neurodegeneration | 42% (121/286) |
| 11 | 51.295 | 49 | 13 | APC,ARNT,Brd4,CD44,ERK1/2 (family),HSPB8,IL6R,LAMA4,LEP,let-7 (includes others),let-7a-5p (and other miRNAs w/seed GAGGUAG),SMAD3,YBX1 | 23 | ACKR3,AGTR1,ALDOA,CXCL8,EDNRB,EPO,GJA1,HGF,ICAM1,IL1R1,ITGB3,JUNB,LDHA,LDLR,LOXL2,MMP19,PDK1,PKM,SOD2,STAT3,THBS1,TNFRSF11B,TPI1 | 13 | Adhesion of blood cells,Adhesion of tumor cell lines,Apoptosis,Binding of leukocytes,Cell movement of tumor cell lines,Cell spreading,Cell viability of myeloid cells,Cell viability of tumor cell lines,Cell-cell contact,Chemotaxis,Inflammation of body cavity,Invasion of cells,Migration of cells | 49% (82/169) |
| 12 | 50.44 | 71 | 16 | CCL5,CD5,CEBPB,COLLAGENASE (family),CSF2,ECSIT,IGE (complex),IKBKB,IL22,IL33,IL5,IL6,JAK1,KLF6,LIF,NFKB (complex) | 39 | ACKR3,ADAMTS4,ALDOA,B4GALT1,BCL3,C1R,CCR1,CXCL8,EDNRB,EGLN3,ELF3,EPO,ETS2,FGF23,HGF,HPSE,ICAM1,IL1R1,ITGB3,JUNB,LDHA,LDLR,MMP19,NDRG1,OSMR,PAPSS2,PDE4B,PDK1,PFKP,PHLDA1,PKM,RGS4,SEMA4A,SLC16A3,SOD2,SORL1,STAT3,THBS1,TNFRSF11B | 16 | Adhesion of tumor cell lines,Advanced malignant tumor,Apoptosis,Cell death of tumor cell lines,Cell proliferation of tumor cell lines,Cell viability,Cellular degradation,Degeneration of cells,Degeneration of nervous system,Differentiation of helper T lymphocytes,Hypoplasia of organ,Invasive cancer,Migration of tumor cell lines,Neurodegeneration,Proliferation of osteoblasts,Synthesis of nucleotide | 39% (99/256) |
| 13 | 48.22 | 59 | 13 | CD3 (complex),EDN1,ERK1/2 (family),F2,FN1,MAPK (family),MEK (family),MMP9,OSCAR,TICAM1,TNFRSF1A,TNFRSF1B,YBX1 | 33 | AGTR1,B4GALT1,BCL3,C1R,CCR1,CLDN5,CXCL8,EDNRB,EPO,ETS2,FGF23,GJA1,HGF,ICAM1,IL1R1,ITGB3,JUNB,LBP,LDLR,LOXL2,MMP19,P2RX7,PDE4B,PDK1,PHLDA1,PKM,SOD2,SORL1,SRGN,STC2,THBS1,TTYH3,VCL | 13 | Activation of myeloid cells,Activation of phagocytes,Adhesion of blood cells,Binding of leukocytes,Cell movement of myeloid cells,Cell spreading,Cell viability of myeloid cells,Cell-cell contact,Cellular homeostasis,Chemotaxis,Inflammation of airway,Interaction of tumor cell lines,Migration of cells | 55% (93/169) |
| 14 | 47.206 | 70 | 15 | AHR,CCL5,CYTOKINE (family),EGF,IL1A,IL6,IL6R,KITLG,KLF6,LEP,PDGF-BB (complex),PDGFB,SP1,TCR (complex),TLR4 | 38 | ACKR3,ADAMTS4,ADORA2A,AGTR1,ALDOA,B4GALT1,BCL3,CCR1,CXCL8,DIO3,EDNRB,EGLN3,ELF3,EPO,FGF23,GJA1,HGF,HPSE,ICAM1,IL1R1,ITGB3,JUNB,LDHA,LDLR,MMP19,NDRG1,P2RX7,PDE4B,PDK1,PHLDA1,PKM,SAMSN1,SEMA4A,SOD2,STAT3,THBS1,TNFRSF11B,VCL | 17 | Cell viability of tumor cell lines,Development of neurons,Differentiation of T lymphocytes,Epithelial-mesenchymal transition,Formation of blood vessel,Hypoplasia of organ,Interaction of tumor cell lines,Migration of smooth muscle cells,Migration of tumor cell lines,Mobilization of cells,Outgrowth of cells,Perinatal death,Polarization of cells,Survival of neural cells,Synthesis of nucleotide,Tubulation of cells,Tubulation of epithelial tissue | 41% (105/255) |
| 15 | 46.96 | 77 | 19 | CD28,CYTOKINE (family),EGF,EPAS1,ERBB2,ERK (family),IKBKB,IL1A,IL1B,IL2,IL6,KITLG,KLF6,P38 MAPK (family),PDGF-BB (complex),SP1,STAT3,TCR (complex),TLR4 | 40 | ACKR3,ADORA2A,AGTR1,ALDOA,B4GALT1,BCL3,CCR1,CLDN5,CXCL8,DIO3,EDNRB,ELF3,EPO,GBE1,GJA1,HGF,HPSE,ICAM1,IHH,IL1R1,ITGB3,JUNB,LBP,LDHA,LDLR,MAFF,P2RX7,PDE4B,PDK1,PHLDA1,PKM,PTGIS,RGS4,SAMSN1,SEMA4A,SOD2,SRGN,THBS1,TNFRSF11B,VCL | 18 | Binding of granulocytes,Cell viability of myeloid cells,Differentiation of T lymphocytes,Formation of blood vessel,Increased Levels of Hematocrit,Interaction of tumor cell lines,Migration of keratinocytes,Migration of smooth muscle cells,Outgrowth of cells,Perinatal death,Polarization of cells,Proliferation of osteoblasts,Regeneration of cells,Sphere formation of carcinoma cell lines,Survival of neural cells,Synthesis of nucleotide,Tubulation of cells,Tubulation of epithelial tissue | 34% (117/342) |
| 16 | 44.978 | 51 | 11 | APC,Brd4,CD44,ERK1/2 (family),F2,HSPB8,let-7 (includes others),MRTFA,RELA,SMAD3,YBX1 | 28 | ACKR3,ALDOA,B4GALT1,BCL3,CXCL8,EDNRB,ELF3,EPO,F11R,GJA1,HGF,ICAM1,ITGB3,JUNB,LDHA,LDLR,LOXL2,MMP19,PDE4B,PDK1,PKM,SEMA3D,SOD2,SRGN,STAT3,THBS1,TNFRSF11B,VCL | 12 | Adhesion of blood cells,Adhesion of tumor cell lines,Binding of leukocytes,Cell movement of myeloid cells,Cell movement of tumor cell lines,Cell spreading,Cell viability of myeloid cells,Cell viability of tumor cell lines,Cell-cell contact,Chemotaxis,Invasion of cells,Migration of cells | 53% (70/132) |
| 17 | 44.735 | 40 | 9 | BHLHE40,CEBPB,EGR1,ERK (family),IKBKB,NPM1,SP1,STAT3,TLR2 | 21 | AGTR1,BAIAP2,CCR1,CXCL8,GJA1,HGF,HPSE,ICAM1,ITGB3,JUNB,LDLR,NDRG1,P2RX7,PHLDA1,PKM,SBNO2,SLC16A3,SLC6A6,SOD2,THBS1,TNFRSF11B | 10 | Adhesion of tumor cell lines,Cell viability of myeloid cells,Cell-cell contact,Cellular degradation,Degeneration of cells,Degeneration of nervous system,Neurodegeneration,Outgrowth of cells,Survival of neural cells,Synthesis of nucleotide | 29% (26/90) |
| 18 | 44.624 | 61 | 13 | AHR,CCL5,IKBKB,IL15,IL22,IL6,IL6R,KITLG,KLF6,LEP,MYC,PDGF-BB (complex),PDGFB | 35 | ACKR3,ADAMTS4,ALDOA,BCL3,CCR1,CXCL8,EDNRB,EGLN3,EPO,ETS2,FGF23,GJA1,HGF,ICAM1,IL1R1,ITGB3,JUNB,LDHA,LDLR,MMP19,NDRG1,PDK1,PFKP,PHLDA1,PKM,SEMA4A,SLC16A3,SOD2,SORL1,SRGN,STAT3,THBS1,TNFRSF11B,TPI1,VCL | 13 | Adhesion of tumor cell lines,Cell death of carcinoma cell lines,Cell viability of tumor cell lines,Development of neurons,Differentiation of T lymphocytes,Epithelial-mesenchymal transition,Glycolysis of cells,Hypoplasia of organ,Migration of smooth muscle cells,Migration of tumor cell lines,Survival of neural cells,Tubulation of cells,Tubulation of epithelial tissue | 43% (73/169) |
| 19 | 39.528 | 74 | 15 | CCL5,CYTOKINE (family),EGF,IL1A,IL6,IL6R,KITLG,KLF6,LEP,PDGF-BB (complex),PDGFB,PIWIL4,SP1,TCR (complex),TLR4 | 40 | ACKR3,ADAMTS4,ADORA2A,AGTR1,ALDOA,B4GALT1,BCL3,CCR1,CXCL8,DIO3,EDNRB,EGLN3,ELF3,EPO,ETS2,FGF23,GJA1,HGF,HPSE,ICAM1,IL1R1,ITGB3,JUNB,LDHA,LDLR,MMP19,NDRG1,P2RX7,PDE4B,PDK1,PHLDA1,PKM,PLSCR1,SAMSN1,SEMA4A,SOD2,STAT3,THBS1,TNFRSF11B,VCL | 19 | Cell viability,Development of neurons,Differentiation of T lymphocytes,Dysgenesis,Epithelial-mesenchymal transition,Formation of blood vessel,Interaction of tumor cell lines,Migration of smooth muscle cells,Migration of tumor cell lines,Mobilization of cells,Outgrowth of cells,Perinatal death,Polarization of cells,Proliferation of neuronal cells,Sphere formation of tumor cell lines,Survival of neural cells,Synthesis of nucleotide,Tubulation of cells,Tubulation of epithelial tissue | 39% (112/285) |
| 20 | 38.249 | 87 | 21 | APC,Brd4,CD44,ERK1/2 (family),FLCN,FN1,HSPB8,IL1A,IL6,IL6R,KITLG,KLF6,LEP,let-7 (includes others),MEK (family),NFKB (complex),NRG1,PDGF-BB (complex),PDGFB,TLR4,YBX1 | 48 | ACKR3,ADAMTS4,ADORA2A,AGTR1,ALDOA,B4GALT1,BCL3,CCR1,CXCL8,EDNRB,EGLN3,ELF3,EPO,ETS2,F13A1,FGF23,GJA1,GP5,HGF,HPSE,ICAM1,IL1R1,ITGB3,JUNB,LBP,LDHA,LDLR,LOXL2,MAFF,MMP19,NDRG1,PDE4B,PDK1,PHLDA1,PKM,PLSCR1,SAMSN1,SBNO2,SEMA4A,SLC16A3,SOD2,SORL1,ST3GAL4,STAT3,THBS1,TNFRSF11B,TTYH3,VCL | 18 | Adhesion of blood cells,Adhesion of tumor cell lines,Binding of leukocytes,Cell spreading,Cell viability,Cell-cell contact,Chemotaxis,Development of neurons,Differentiation of T lymphocytes,Dysgenesis,Invasion of cells,Migration of smooth muscle cells,Migration of tumor cell lines,Organismal death,Polarization of cells,Survival of neural cells,Tubulation of cells,Tubulation of epithelial tissue | 51% (193/378) |
| 21 | 34.497 | 43 | 8 | ARNT,GNAS,HISTONE DEACETYLASE (complex),LAMA4,LEP,let-7 (includes others),let-7a-5p (and other miRNAs w/seed GAGGUAG),SMAD3 | 24 | AGTR1,ALDOA,CXCL8,EPO,ETS2,HGF,HPSE,ICAM1,IL1R1,ITGB3,JUNB,LDHA,LDLR,LOXL2,MAFF,MMP19,PDE4B,PKM,PTGIS,SOD2,STAT3,THBS1,TNFRSF11B,TPI1 | 11 | Adhesion of blood cells,Adhesion of tumor cell lines,Apoptosis,Binding of leukocytes,Cell movement of tumor cell lines,Cell viability of tumor cell lines,Chemotaxis,Inflammation of body cavity,Invasion of cells,Migration of cells,Survival of organism | 52% (46/88) |
| 22 | 32.667 | 58 | 12 | CCL5,CSF2,GPR174,IFNG,IKBKB,IL22,IL6,KLF6,miR-34a-5p (and other miRNAs w/seed GGCAGUG),PDGFB,RRAS2,TCR (complex) | 36 | ADAMTS4,ADORA2A,AGTR1,ALDOA,ARG2,BCL3,CXCL8,EDNRB,EGLN3,EPO,ETS2,F11R,GJA1,HGF,ICAM1,IL1R1,ITGB3,JUNB,LDHA,LDLR,MMP19,MPZL2,OSMR,P2RX7,PDE4B,PHLDA1,PKM,PLSCR1,SEMA4A,SLC6A6,SOD2,SORL1,STAT3,THBS1,TNFRSF11B,VCL | 10 | Adhesion of tumor cell lines,Apoptosis,Cell death of carcinoma cell lines,Cell viability of blood cells,Development of neurons,Epithelial-mesenchymal transition,Leukopoiesis,Migration of smooth muscle cells,Migration of tumor cell lines,Proliferation of osteoblasts | 42% (50/120) |
| 23 | 31.435 | 44 | 10 | ERK (family),IKBKB,IL1B,IL6,KITLG,P38 MAPK (family),PDGF-BB (complex),SP1,STAT3,TLR4 | 24 | ADORA2A,AGTR1,ALDOA,CCR1,CXCL8,EDNRB,EPO,GJA1,HGF,HPSE,ICAM1,IL1R1,ITGB3,JUNB,LBP,P2RX7,PHLDA1,PKM,PTGIS,RGS4,SOD2,SRGN,THBS1,TNFRSF11B | 10 | Adhesion of tumor cell lines,Binding of granulocytes,Cell viability of myeloid cells,Mobilization of cells,Outgrowth of cells,Proliferation of osteoblasts,Survival of neural cells,Synthesis of purine nucleotide,Tubulation of cells,Tubulation of epithelial tissue | 45% (45/100) |
| 24 | 31.423 | 40 | 7 | CD3 (complex),EDN1,ERK1/2 (family),FN1,KITLG,STAT4,YBX1 | 21 | BCL3,CCR1,CXCL8,EDNRB,EPO,GJA1,HGF,ICAM1,IL1R1,ITGB3,JUNB,LDLR,PDE4B,PDK1,PKM,SAMSN1,SRGN,ST3GAL4,STAT3,THBS1,VCL | 12 | Adhesion of blood cells,Binding of leukocytes,Cell spreading,Cell viability of blood cells,Cell-cell contact,Chemotaxis,Interaction of tumor cell lines,Migration of endothelial cells,Migration of keratinocytes,Migration of phagocytes,Mobilization of cells,Recruitment of phagocytes | 48% (40/84) |

| | | | | | | | | | |
|---|---|---|---|---|---|---|---|---|---|
| 25 | 30.125 | 53 | 12 | AHR,CCL5,EGF,HBEGF,IL22,IL6,IL6R,KITLG,KLF6,LEP,PDGF-BB (complex),PSME3 | 30 | ACKR3,ALDOA,B4GALT1,BCL3,CCR1,CXCL8,EDNRB,EGLN3,ELF3,EPO,ETS2,FGF23,GJA1,HGF,ICAM1,ITGB3,JUNB,LDHA,LDLR,MMP19,PFKP,PHLDA1,PKM,SEMA4A,SLC16A3,SOD2,STAT3,THBS1,TNFRSF11B,VCL | 11 | Adhesion of tumor cell lines,Cell death of carcinoma cell lines,Cell viability of tumor cell lines,Epithelial-mesenchymal transition,Glycolysis of tumor cell lines,Hypoplasia of organ,Migration of smooth muscle cells,Migration of tumor cell lines,Survival of neural cells,Tubulation of cells,Tubulation of epithelial tissue | 42% (56/132) |
| 26 | 30.061 | 39 | 11 | CG (complex),IL1A,IL1B,IL2,IL4,IL6,KLF6,SP1,STAT3,TLR4,TNF | 22 | ADORA2A,ALDOA,BCL3,CXCL8,ELF3,HGF,HPSE,ICAM1,IHH,IL1R1,JUNB,LDHA,MMP19,P2RX7,PDE4B,PDK1,PHLDA1,PKM,SAMSN1,SEMA4A,SOD2,SRGN | 6 | Adhesion of colorectal cancer cell lines,Development of carcinoma cell lines,Differentiation of T lymphocytes,Migration of keratinocytes,Polarization of cells,Sphere formation of carcinoma cell lines | 35% (23/66) |
| 27 | 28.503 | 66 | 12 | AGT,CYTOKINE (family),EGF,IL1A,IL1B,IL6,KITLG,KLF6,PDGF-BB (complex),SP1,TCR (complex),TLR4 | 39 | ACKR3,ADAMTS4,ADORA2A,AGTR1,ALDOA,B4GALT1,CCR1,CXCL8,DIO3,EDNRB,EPO,FGF23,GJA1,HGF,HPSE,ICAM1,IL1R1,ITGB3,JUNB,LBP,LDHA,LDLR,LOXL2,NDRG1,P2RX7,PDE4B,PDK1,PHLDA1,PKM,PTGIS,RGS4,SAMSN1,SEMA3D,SOD2,SRGN,STAT3,THBS1,TNFRSF11B,VCL | 15 | Adhesion of immune cells,Atrophy of kidney,Contraction of cells,Epithelial-mesenchymal transition,Formation of blood vessel,Interaction of tumor cell lines,Migration of smooth muscle cells,Mobilization of cells,Outgrowth of cells,Perinatal death,Polarization of cells,Survival of neural cells,Synthesis of nucleotide,Tubulation of cells,Tubulation of epithelial tissue | 40% (72/180) |
| 28 | 28.241 | 43 | 11 | ERK (family),HBEGF,HIF1A,IKBKB,IL15,IL6,KITLG,KLF6,MYC,P38 MAPK (family),PDGF-BB (complex) | 26 | ACKR3,ALDOA,CXCL8,EDNRB,EGLN3,ELF3,EPO,HGF,ICAM1,IL1R1,ITGB3,JUNB,LBP,LDHA,PDK1,PFKP,PHLDA1,PKM,PTGIS,RGS4,SLC16A3,SOD2,SRGN,STAT3,THBS1,TPI1 | 6 | Adhesion of tumor cell lines,Binding of granulocytes,Cell viability of tumor cell lines,Glycolysis of cells,Tubulation of cells,Tubulation of epithelial tissue | 41% (27/66) |
| 29 | 28.134 | 36 | 5 | CEBPB,IL22,LCN2,LIF,PIK3R1 | 17 | BCL3,CCR1,CXCL8,ELF3,HGF,HPSE,ICAM1,JUNB,LBP,LDHA,LDLR,PDK1,PKM,RGS4,SOD2,STAT3,TNFRSF11B | 14 | Advanced malignant tumor,Apoptosis,Cell death of tumor cell lines,Cell viability,Cellular degradation,Colony formation of cells,Degeneration of cells,Degeneration of nervous system,Invasion of cells,Invasive cancer,Liver lesion,Migration of tumor cell lines,Neurodegeneration,Survival of organism | 67% (47/70) |
| 30 | 27.483 | 47 | 6 | EGF,IL1B,IL6,KITLG,PDGF-BB (complex),SP1 | 29 | ACKR3,ADAMTS4,AGTR1,B4GALT1,CCR1,CXCL8,DIO3,EDNRB,EPO,ETS2,FGF23,GJA1,HGF,HPSE,ICAM1,IL1R1,ITGB3,JUNB,LDHA,LDLR,P2RX7,PDE4B,PKM,PTGIS,RGS4,SOD2,STAT3,THBS1,TNFRSF11B | 12 | Atrophy of kidney,Binding of protein binding site,Flux of Ca2 ,Formation of blood vessel,Migration of smooth muscle cells,Mobilization of cells,Outgrowth of cells,Perinatal death,Survival of neural cells,Synthesis of purine nucleotide,Tubulation of cells,Tubulation of epithelial tissue | 54% (39/72) |
| 31 | 27.4 | 55 | 9 | AGT,EDN1,ERK1/2 (family),FN1,KITLG,MEK (family),NRG1,PDGF-BB (complex),YBX1 | 32 | ACKR3,ADAMTS4,AGTR1,BCL3,CCBE1,CXCL8,EDNRB,EPO,ETS2,FGF23,GJA1,HGF,ICAM1,IL1R1,ITGB3,JUNB,LBP,LDLR,LOXL2,MAFF,NDRG1,PHLDA1,PKM,SAMSN1,SEMA3D,SOD2,ST3GAL4,STAT3,THBS1,TNFRSF11B,TTYH3,VCL | 14 | Adhesion of blood cells,Adhesion of tumor cell lines,Binding of leukocytes,Cell spreading,Cell viability of myeloid cells,Cell-cell contact,Chemotaxis,Contraction of cells,Migration of endothelial cells,Migration of keratinocytes,Migration of phagocytes,Organismal death,Tubulation of cells,Tubulation of epithelial tissue | 52% (65/126) |
| 32 | 27.353 | 39 | 6 | ECSIT,FN1,KITLG,MEK (family),PDGF-BB (complex),SMAD3 | 24 | ACKR3,BCL3,CXCL8,EDNRB,EPO,ETS2,GJA1,ICAM1,IL1R1,ITGB3,JUNB,LBP,LDLR,MAFF,MMP19,PHLDA1,PKM,SOD2,ST3GAL4,STAT3,THBS1,TNFRSF11B,TTYH3,VCL | 9 | Adhesion of blood cells,Adhesion of tumor cell lines,Binding of leukocytes,Chemotaxis,Inflammation of body cavity,Organismal death,Survival of organism,Tubulation of cells,Tubulation of epithelial tissue | 43% (23/54) |
| 33 | 27.315 | 38 | 7 | AGT,CD28,CD3 (complex),EDN1,FN1,MEK (family),OSCAR | 23 | AGTR1,C1R,CCR1,CXCL8,EDNRB,GJA1,HGF,ICAM1,IL1R1,ITGB3,JUNB,LDLR,MMP19,NDRG1,PDE4B,PHLDA1,SEMA3D,SOD2,SRGN,STAT3,THBS1,TNFRSF11B,VCL | 8 | Adhesion of blood cells,Adhesion of tumor cell lines,Binding of leukocytes,Cell spreading,Chemotaxis,Contraction of cells,Inflammation of airway,Leukocyte migration | 54% (30/56) |
| 34 | 26.74 | 40 | 6 | HIF1 (complex),IL22,KLF6,PALMD,PIK3R1,VEGF (family) | 24 | ACKR3,ADORA2A,ALDOA,BCL3,CCR1,CXCL8,EPO,ETS2,HPSE,ICAM1,IL1R1,ITGB3,JUNB,LDHA,LOXL2,PDK1,PFKP,PHLDA1,PKM,PLOD2,SLC16A3,SOD2,STAT3,TNFRSF11B | 10 | Advanced malignant tumor,Cell death of carcinoma cell lines,Cell proliferation of tumor cell lines,Cell viability of blood cells,Glycolysis of tumor cell lines,Invasion of cells,Invasive cancer,Migration of endothelial cells,Migration of tumor cell lines,Proliferation of neural cells | 43% (26/60) |
| 35 | 23.708 | 55 | 11 | HIF1A,IL1A,IL1B,IL2,IL6,KLF6,SKIC2,SP1,STAT3,TLR4,TNF | 31 | ACKR3,ADAMTS4,ADORA2A,ALDOA,BCL3,CXCL8,ELF3,EPO,HGF,HPSE,ICAM1,IL1R1,ITGB3,JUNB,LDHA,LOXL2,NDRG1,P2RX7,PDE4B,PDK1,PFKP,PHLDA1,PKM,PLOD2,RGS4,SEMA4A,SLC16A3,SOD2,SRGN,THBS1,TPI1 | 13 | Adhesion of colorectal cancer cell lines,Branching of cells,Development of carcinoma cell lines,Differentiation of helper T lymphocytes,Epithelial-mesenchymal transition of tumor cell lines,Formation of blood vessel,Glycolysis of cells,Inflammation of muscle,Invasion of tumor cells,Migration of keratinocytes,Polarization of cells,Sphere formation of carcinoma cell lines,Sprouting | 41% (58/143) |
| 36 | 23.665 | 37 | 6 | AGT,EDN1,FN1,HIF1A,IL6,KITLG | 22 | AGTR1,CCR1,CXCL8,EDNRB,EPO,GP5,HGF,ICAM1,IL1R1,ITGB3,JUNB,LBP,LDLR,PDK1,PKM,SAMSN1,SEMA3D,ST3GAL4,STAT3,THBS1,TNFRSF11B,VCL | 9 | Adhesion of blood cells,Adhesion of tumor cell lines,Binding of leukocytes,Cell spreading,Cell viability of lung cancer cell lines,Chemotaxis,Contraction of cells,Tubulation of cells,Tubulation of epithelial tissue | 59% (32/54) |
| 37 | 21.361 | 48 | 9 | IL17A,IL1A,IL1B,IL6,KITLG,KLF6,PDGF-BB (complex),SP1,TLR4 | 30 | ADORA2A,AGTR1,ALDOA,BCL3,CCR1,CXCL8,EDNRB,ELF3,EPO,GJA1,HGF,HPSE,ICAM1,IL1R1,ITGB3,JUNB,LDHA,P2RX7,PDE4B,PDK1,PHLDA1,PKM,PTGIS,RGS4,SAMSN1,SEMA4A,SOD2,SRGN,STAT3,THBS1 | 9 | Adhesion of tumor cell lines,Differentiation of T lymphocytes,Outgrowth of cells,Polarization of cells,Sphere formation of carcinoma cell lines,Survival of neural cells,Synthesis of purine nucleotide,Tubulation of cells,Tubulation of epithelial tissue | 40% (32/81) |
| 38 | 20.984 | 44 | 7 | AHR,CD38,EGF,IL15,IL6,KITLG,PDGF-BB (complex) | 29 | ACKR3,ALDOA,B4GALT1,BCL3,CCR1,CXCL8,EDNRB,EGLN3,ELF3,EPO,FGF23,GJA1,HGF,ITGB3,LDHA,LDLR,NDRG1,PDK1,PFKP,PHLDA1,PKM,SLC16A3,SOD2,SORL1,SRGN,STAT3,THBS1,TNFRSF11B,TPI1 | 8 | Cell viability of tumor cell lines,Epithelial-mesenchymal transition,Glycolysis of cells,Hypoplasia of organ,Migration of smooth muscle cells,Mobilization of cells,Tubulation of cells,Tubulation of epithelial tissue | 34% (19/56) |
| 39 | 20.41 | 41 | 5 | AHR,IL1B,KITLG,PDGF-BB (complex),SP1 | 28 | ACKR3,AGTR1,ALDOA,CCR1,CXCL8,EDNRB,EGLN3,ELF3,EPO,GJA1,HGF,HPSE,ICAM1,IL1R1,ITGB3,JUNB,LBP,LDLR,METRNL,P2RX7,PDE4B,PKM,PTGIS,RGS4,SOD2,STAT3,THBS1,TNFRSF11B | 8 | Inflammation of body cavity,Migration of smooth muscle cells,Mobilization of cells,Outgrowth of cells,Survival of neural cells,Synthesis of purine nucleotide,Tubulation of cells,Tubulation of epithelial tissue | 40% (16/40) |
| 40 | 20.076 | 36 | 8 | AGT,EDN1,IL1 (family),IL1A,IL6,PDGF-BB (complex),STAT3,TLR4 | 21 | ACKR3,ADORA2A,AGTR1,ALDOA,CCR1,CXCL8,EDNRB,GJA1,HGF,ICAM1,ITGB3,JUNB,LDHA,LDLR,PDK1,PHLDA1,PKM,SEMA4A,SOD2,THBS1,VCL | 7 | Cell spreading,Contraction of cells,Interaction of tumor cell lines,Migration of keratinocytes,Migration of phagocytes,Migration of smooth muscle cells,Polarization of cells | 48% (27/56) |
| 41 | 20.017 | 35 | 6 | HIF1A,IL6,KLF6,SP1,TNF,YAP1 | 23 | ACKR3,ALDOA,BCL3,ELF3,EPO,HGF,HPSE,ICAM1,IL1R1,ITGB3,JUNB,LDHA,LOXL2,P2RX7,PDK1,PFKP,PHLDA1,PKM,SEMA4A,SLC16A3,STAT3,THBS1,TPI1 | 6 | Adhesion of colorectal cancer cell lines,Development of carcinoma cell lines,Differentiation of helper T lymphocytes,Formation of blood vessel,Glycolysis of cells,Invasion of tumor cells | 42% (15/36) |
| 42 | 19.392 | 35 | 6 | EPAS1,ERK (family),IKBKB,IL6,P38 MAPK (family),STAT3 | 23 | BCL3,CCR1,CXCL8,EGLN3,EPO,ETS2,GBE1,GJA1,HGF,ICAM1,IL1R1,ITGB3,JUNB,LBP,LDHA,NDRG1,PDK1,PHLDA1,PLOD2,PLSCR1,SOD2,THBS1,TNFRSF11B | 6 | Binding of granulocytes,Cell death of tumor cell lines,Cell viability,Interaction of tumor cell lines,Proliferation of osteoblasts,Regeneration of cells | 53% (19/36) |
| 43 | 18.141 | 39 | 9 | CYTOKINE (family),IL1A,KITLG,KLF6,NPM1,PIWIL4,TLR2,TLR4,VEGF (family) | 23 | ACKR3,ADORA2A,ALDOA,BCL3,CCR1,CXCL8,EPO,GJA1,HPSE,ICAM1,ITGB3,JUNB,LDHA,LDLR,LOXL2,MMP19,PHLDA1,PKM,SAMSN1,SLC16A3,SOD2,STAT3,TNFRSF11B | 7 | Cell viability of blood cells,Cell-cell contact,Interaction of tumor cell lines,Migration of endothelial cells,Mobilization of cells,Polarization of cells,Synthesis of nucleotide | 33% (21/63) |
| 44 | 17.513 | 42 | 5 | EGF,KITLG,PDGF-BB (complex),PDGFB,TLR4 | 27 | ACKR3,ADORA2A,ALDOA,B4GALT1,BCL3,CXCL8,DIO3,EDNRB,EPO,ETS2,GJA1,HGF,HPSE,ICAM1,IL1R1,ITGB3,JUNB,LBP,LDHA,LDLR,PKM,SOD2,ST3GAL4,STAT3,THBS1,TNFRSF11B,VCL | 10 | Adhesion of blood cells,Binding of leukocytes,Binding of protein binding site,Flux of Ca2 ,Migration of smooth muscle cells,Mobilization of cells,Organismal death,Proliferation of neuronal cells,Tubulation of cells,Tubulation of epithelial tissue | 58% (29/50) |
| 45 | 17.146 | 40 | 9 | AGT,CD3 (complex),CD40LG,EDN1,FN1,IL2,MEK (family),P38 MAPK (family),TLR2 | 24 | AGTR1,ALDOA,ARG2,C1R,CCR1,CXCL8,EDNRB,HGF,ICAM1,IL1R1,ITGB3,JUNB,LBP,LDLR,PDE4B,PHLDA1,SAMSN1,SEMA3D,SOD2,SRGN,STAT3,THBS1,TPI1,VCL | 7 | Adhesion of tumor cell lines,Binding of granulocytes,Cell spreading,Chemotaxis,Contraction of cells,Inflammation of airway,Recruitment of leukocytes | 49% (31/63) |
| 46 | 13.64 | 33 | 4 | BHLHE40,CD5,CEBPB,IL22 | 20 | BCL3,CCR1,CXCL8,EGLN3,GBE1,HGF,ICAM1,LBP,LDHA,LDLR,MAFF,NDRG1,PDE4B,PHLDA1,PLSCR1,RGS4,SLC16A3,SLC6A6,SOD2,STAT3 | 9 | Cell death of tumor cell lines,Cell viability of blood cells,Cellular degradation,Degeneration of cells,Degeneration of nervous system,Differentiation of helper T lymphocytes,Migration of tumor cell lines,Neurodegeneration,Transport of molecule | 28% (10/36) |
| 47 | 3.5 | 7 | 1 | LCN2 | 4 | CCR1,LDLR,SOD2,TNFRSF11B | 2 | Inflammation of organ,Survival of organism | 100% (2/2) |
| 48 | -4.491 | 8 | 1 | LEP | 6 | AGTR1,FGF23,IL1R1,SOD2,STAT3,TNFRSF11B | 1 | Size of body | 100% (1/1) |
| 49 | -5.307 | 8 | 1 | TAFAZZIN | 6 | LDHA,LOXL2,PKM,PLOD2,RGS4,THBS1 | 1 | Metastasis | 0% (0/1) |
| 50 | -14.758 | 9 | 1 | miR-122-5p (miRNAs w/seed GGAGUGU) | 5 | ALDOA,EGLN3,OSMR,PKM,TTYH3 | 3 | Apoptosis,Migration of cells,Necrosis | 100% (3/3) |

https://resources.qiagenbioinformatics.com/white-papers/Regulator_Effects_in_IPA.pdf
