# Supplementary figures and images for: Linking epidemiology and genomics of maternal smoking during pregnancy in utero and in ageing: a population-based study using human foetuses and the UK Biobank cohort
Source: eBioMedicine. 2025 Mar 12;114:105590. doi: 10.1016/j.ebiom.2025.105590 (PMC12121433; doi:10.1016/j.ebiom.2025.105590)

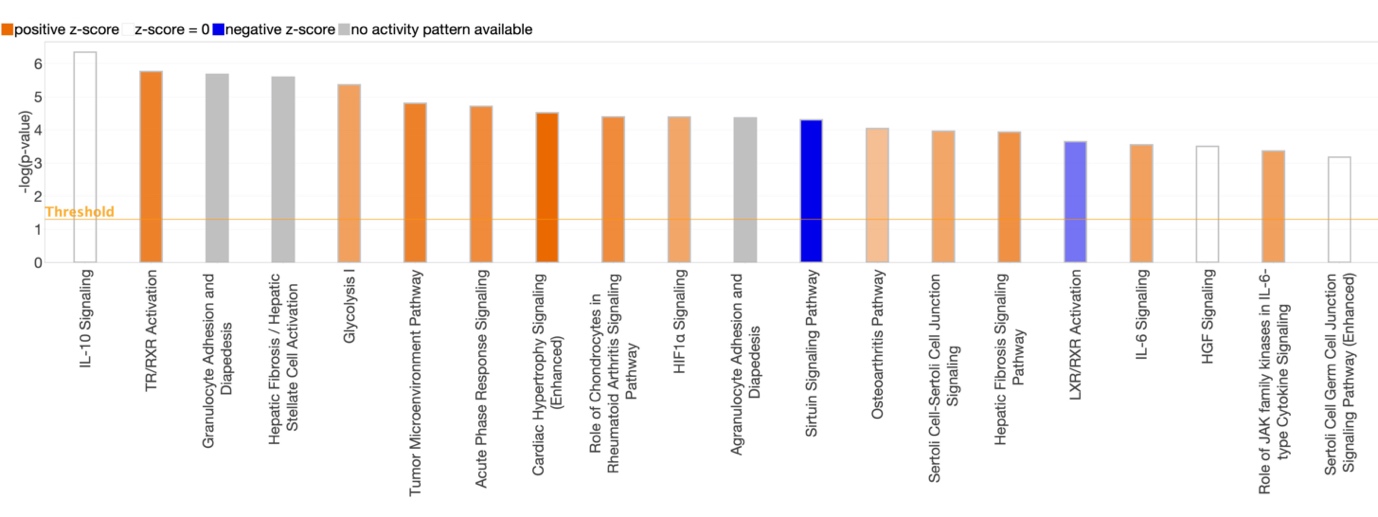

Supplement: Supplementary Figure S1 — Top 20 canonical pathways associated with maternal smoking in the late gestational window male foetal livers. Associations were picked based on z-score, p-value and at least 2-fold activation (positive z-score, 12 pathways) or inhibition (negative z-score, 2 pathways). A preponderance of activation across key pathways was observed. Three of the 20 pathways had z-scores of zero and 3 had no available activity pattern. [file mmc5.docx]

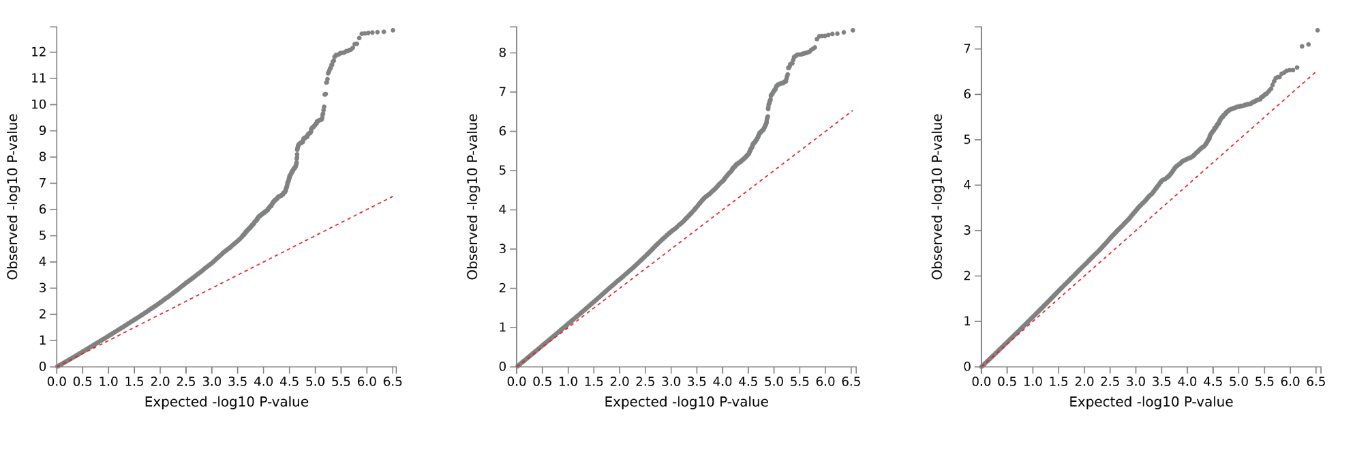

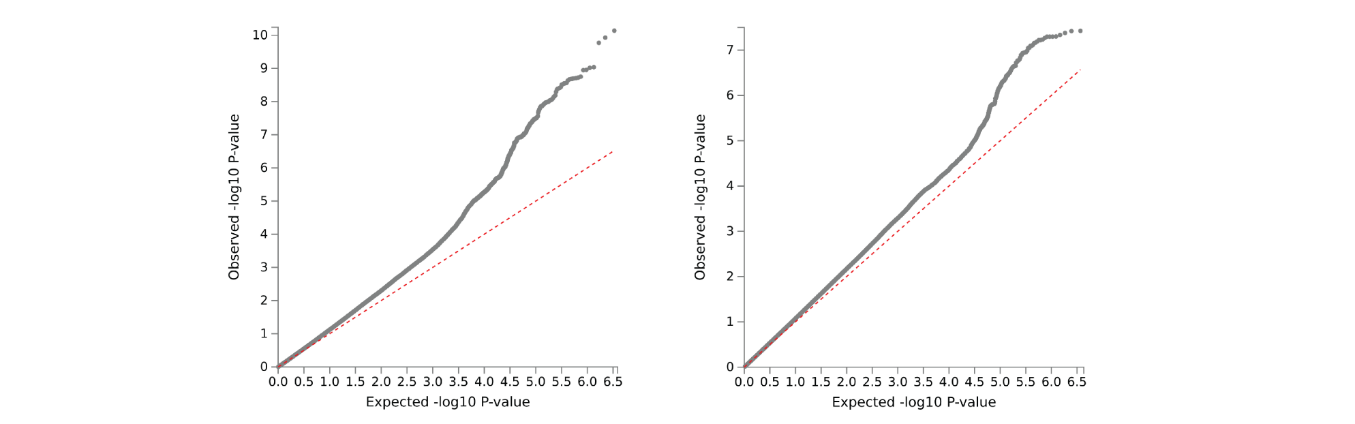


**a**

**b**

**c**

**e**

**d**

Supplement: Supplementary Figure S2 — Quantile-quantile plot of the observed versus expected (theoretical) GWAS values for (a) overall cohort, (b) females, (c) males, (d) non-smokers, (e) smokers. Red line represents the expected (theoretical) distribution of P-values. Common alleles are usually non-significant and follow the line whereas rare significant alleles tend to deviate from the expectation. [file mmc6.docx]

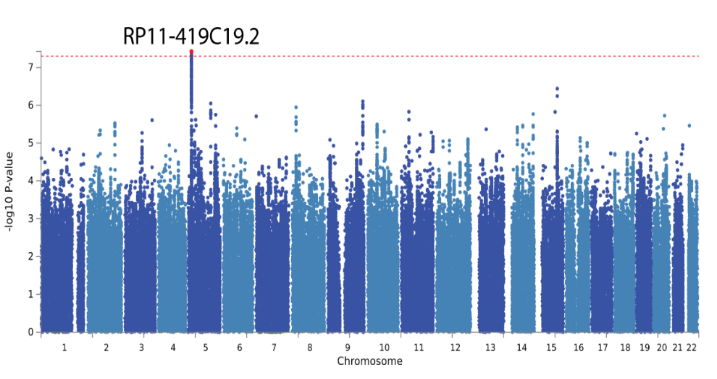

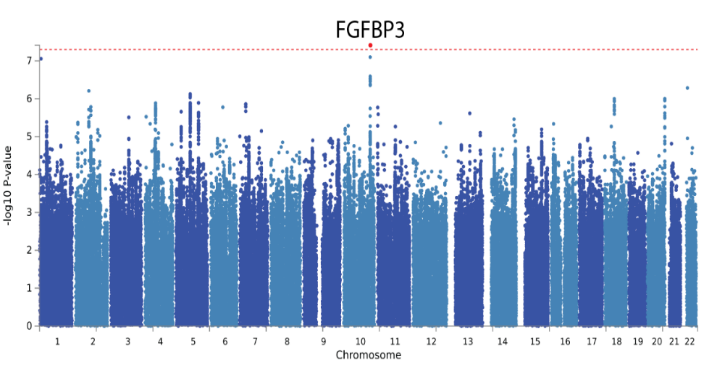

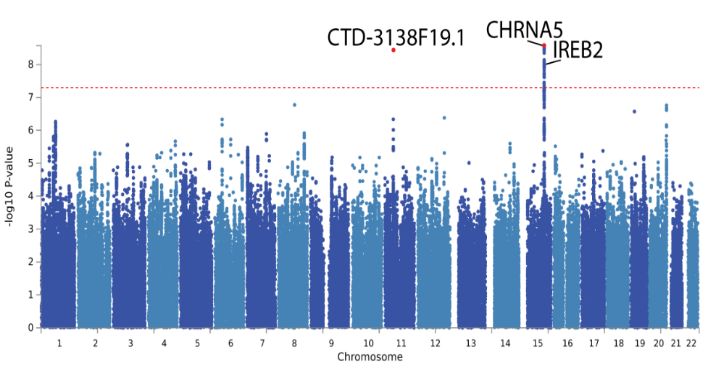

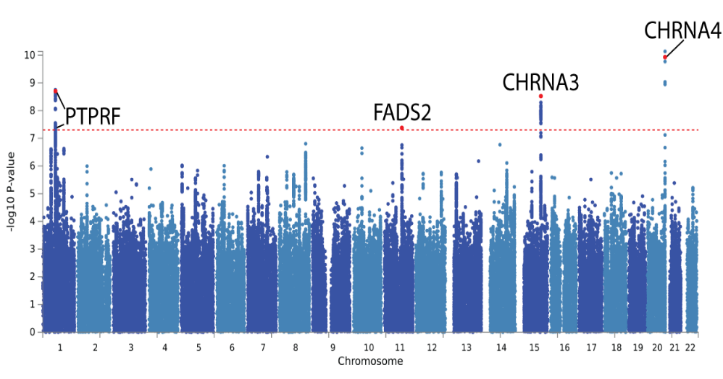


**a**

**b**

**d**

**c**

Supplement: Supplementary Figure S3 — Summary of the genome-wide association studies of maternal smoking for females (a), males (b), smokers (c), and non-smokers (d). Manhattan plots of (a) 4,954,542; (b) 4,925,554; (c) 5,374,679; and (d) 4,864,215 short nucleotide polymorphisms with minor allele frequency of at least 1%. Only genes harbouring exonic variants have been labelled. Red dots indicate genes in each region harbouring a statistically significant variant which has a CADD score greater than 12.37, indicating it is likely influencing that gene's function. The red line denotes genome-wide significance (P < 5 × 10^–8^, logistic regression). The estimated genomic inflation factors λ were (a) 1.14, (b) 1.135, (c) 1.109, (d) 1.152. [file mmc7.docx]

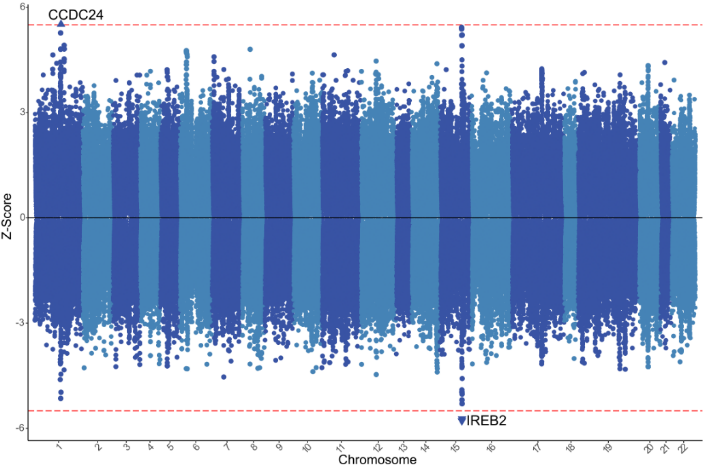

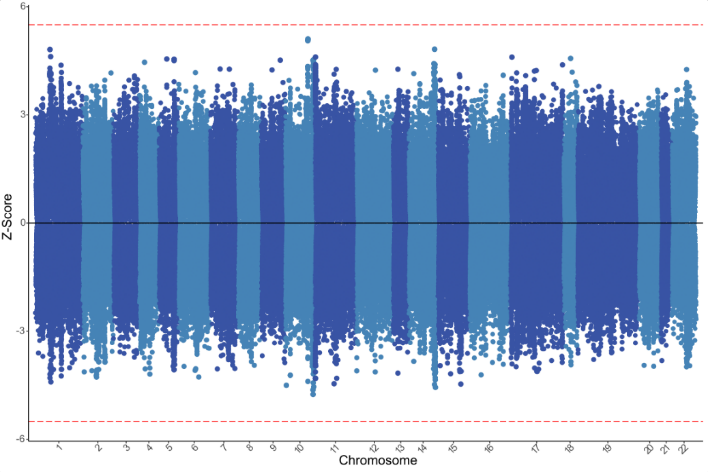

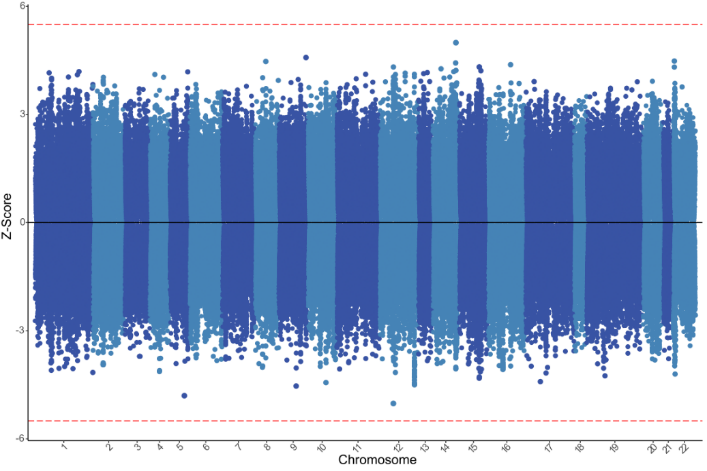

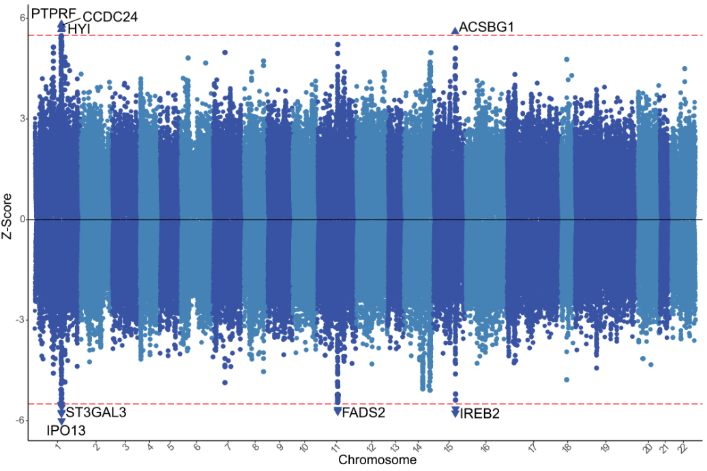


**a**

**b**

**d**

**c**

Supplement: Supplementary Figure S4 — Summary of the transcriptome-wide association studies of maternal smoking for (a) females, (b) males, (c) smokers, (d) non-smokers. Manhattan plots representing the association of GWAS variants with GTEx expression panels from 72 tissues. Only known coding genes reaching statistical significance are labelled. The red line denotes genome-wide significance (|Z-score| > 5.5). Direction of the triangles indicates genes predicted to be overexpressed (facing up) or underexpressed (facing down). [file mmc8.docx]

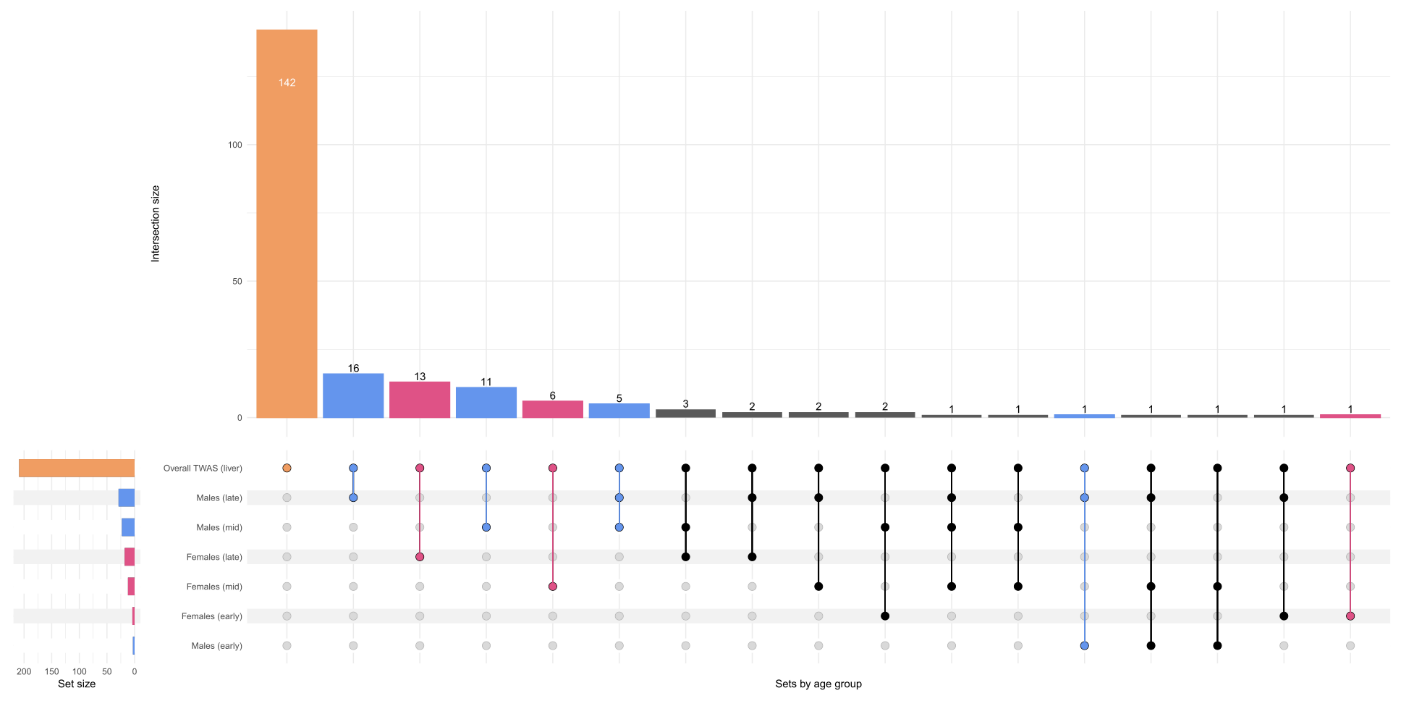

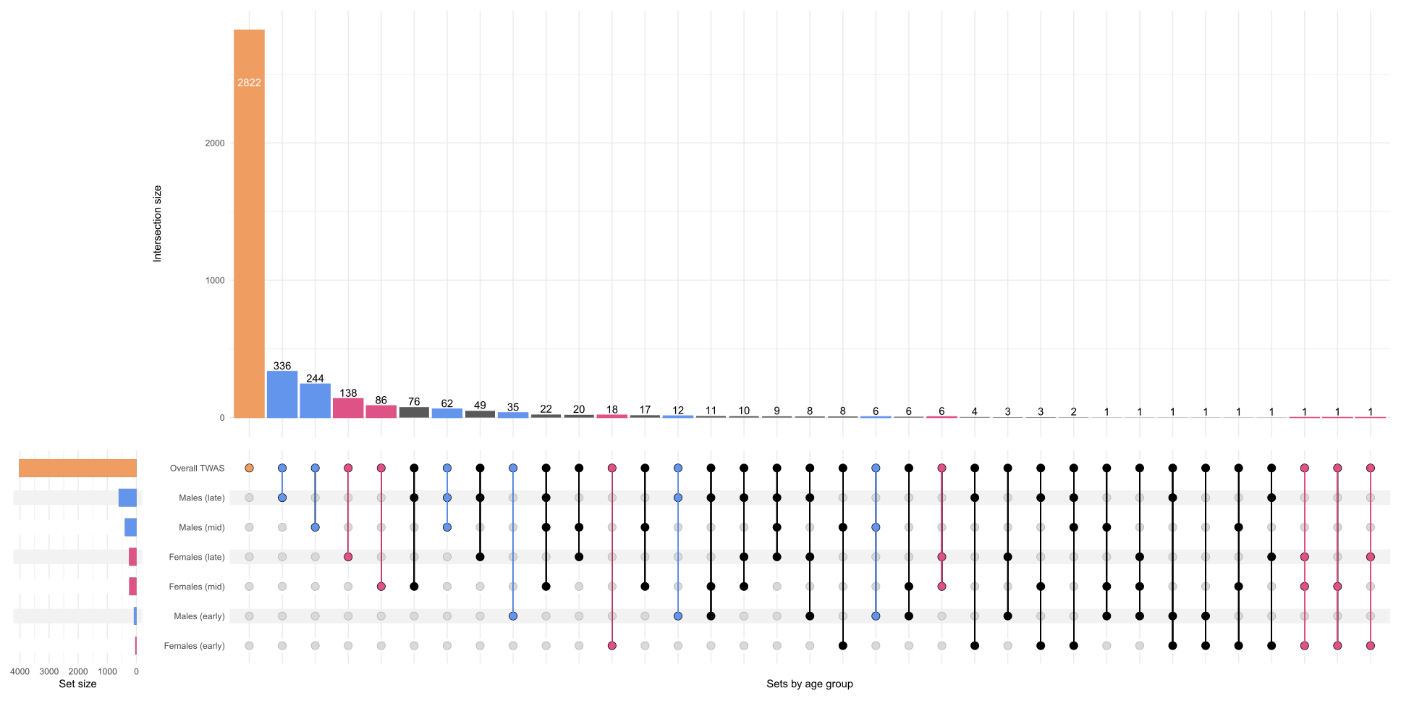


**a**

**b**

Supplement: Supplementary Figure S5 — Upset plot representing the number of unique and overlapping genes between the differential gene expression in foetal livers from males and females at early (11–13), mid (14–16), late (17–19) weeks of gestation and overall (a) and liver specific (b) TWAS. Set size represents the total number genes considered for each category. Intersection size represents the number of genes in the corresponding intersection. Connected dots in the intersection indicate overlap in DE and/or TWAS genes whereas solo dots indicate intersections with genes unique for the category. DE genes were considered at P < 0.05 or |Log Fold Change| > 0.5. TWAS genes were considered at P < 0.01 (logistic regression). Only intersections with a maximum of four overlapping category sets were considered. [file mmc9.docx]
