# Supplementary material for: Linking epidemiology and genomics of maternal smoking during pregnancy in utero and in ageing: a population-based study using human foetuses and the UK Biobank cohort
Source: eBioMedicine. 2025 Mar 12;114:105590. doi: 10.1016/j.ebiom.2025.105590 (PMC12121433; doi:10.1016/j.ebiom.2025.105590)
Supplement: Supplementary Figure S6 [file mmc10.docx]

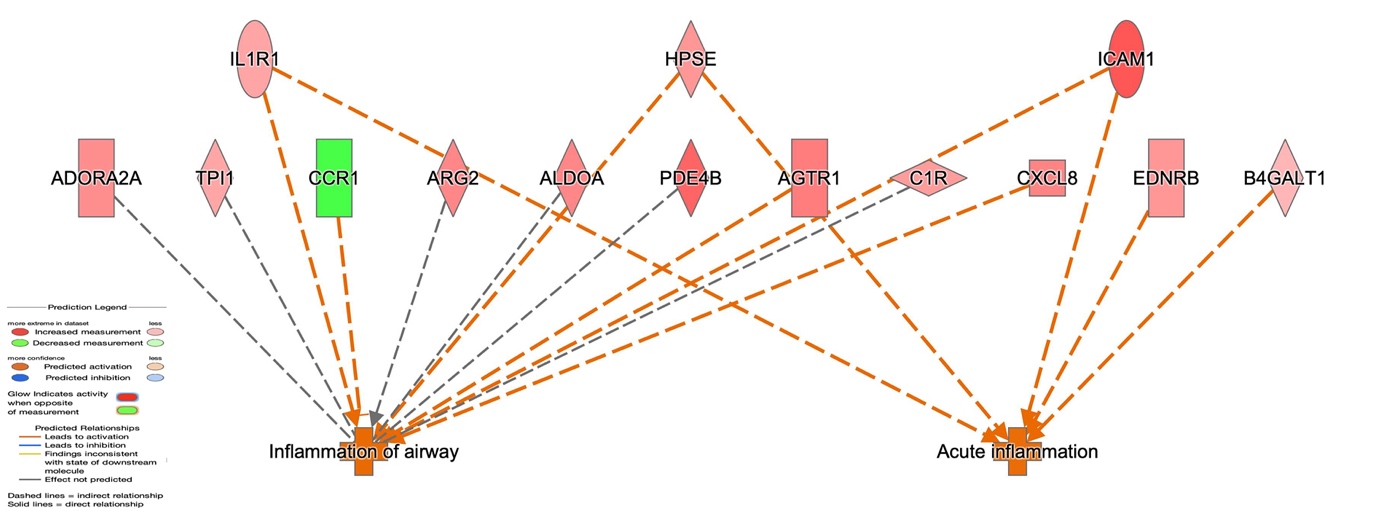

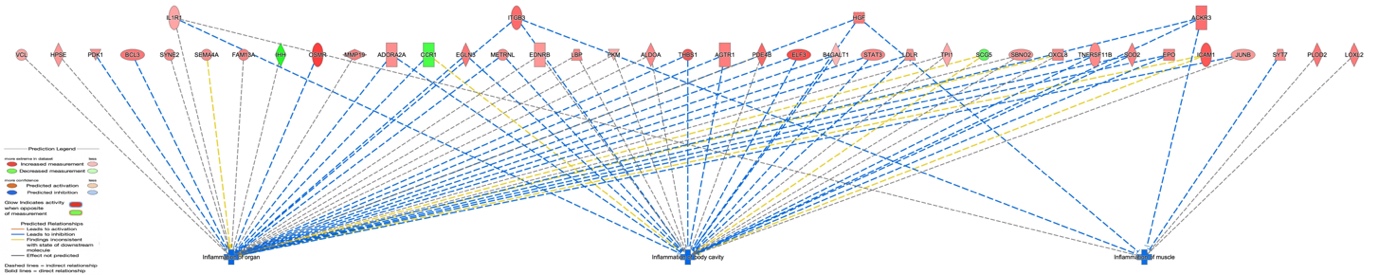


**b**

**a**

**Supplementary Figure 6.** **Two disease networks taken from Disease and Biological function pathways (Supplementary Table 2).** **a)** Stimulation of immune system, combining two pathways; **b)** inhibition of the immune system combining 3 pathways.
